# Testing the power-law hypothesis of the interconflict interval

**DOI:** 10.1038/s41598-023-50002-w

**Published:** 2023-12-19

**Authors:** Hiroshi Okamoto, Iku Yoshimoto, Sota Kato, Budrul Ahsan, Shuji Shinohara

**Affiliations:** 1 Department of Bioengineering, School of Engineering, The University of Tokyo, Tokyo, Japan; 2 Department of Advanced Social and International Studies, Graduate School of Arts and Sciences, The University of Tokyo, Tokyo, Japan; 3 The Tokyo Foundation for Policy Research, Tokyo, Japan; 4 School of Science and Engineering, Tokyo Denki University, Saitama, Japan

**Keywords:** Statistics, Information theory and computation

## Abstract

War is an extreme form of collective human behaviour characterized by coordinated violence. We show that this nature of war is substantiated in the temporal patterns of conflict occurrence that obey power law. The focal metric is the interconflict interval (ICI), the interval between the end of a conflict in a dyad (i.e. a pair of states) and the start of the subsequent conflict in the same dyad. Using elaborate statistical tests, we confirmed that ICI samples compiled from the history of interstate conflicts from 1816 to 2014 followed a power-law distribution. We then demonstrate that the power-law properties of ICIs can be explained by a hypothetical model assuming an information-theoretic formulation of the Clausewitz thesis on war: the use of force is a means of interstate communication. Our findings help us to understand the nature of wars between regular states, the significance of which has increased since the Russian invasion of Ukraine in 2022.

## Introduction

War is an extreme form of collective human behaviour characterized by two seemingly contradictory properties: violence and coordination. War is obviously an act of violence. It kills and injures people, destroying lands and facilities. By contrast, the use of violence in war is not necessarily haphazard. It is coordinated under the leadership of political/military organizations to advance certain objectives. International relations theory is a discipline to study various events that occur in the international community. Whether one stands in any school of thought, such as realism or liberalism, understanding the causes of war is a central issue in international relations theory^1^.

Finding statistical patterns of war is pivotal as it promotes an inductive approach to wars. This approach, which has been reliably employed in the natural sciences, is meant to formulate a hypothesis to account for experimental or empirical observations, and then test predictions from this hypothesis. In international relations theory, in contrast, the theory-based approach, which attempts to derive a novel theory from plausible assumptions by deduction, has been more favoured for solving, for instance, *war’s inefficiency puzzle*^2^.

Robust statistical patterns, if found, can also be utilized to forecast conflict occurrences^3^. Forecasting conflicts is useful not only for scholarly evaluation of theories but also for supporting policymaking by international organizations to prevent, manage, or resolve conflicts or by individual states to establish national security. From this practical perspective, finding robust statistical patterns in any aspect of conflict is desirable.

Notably, scholars in complexity science have more enthusiastically sought statistical patterns of conflicts than scholars in the mainstream of international relations theory. The English physicist Lewis Fry Richardson made outstanding findings more than three-quarters of a century ago^4,5^. He found a power-law relationship between the severity of war, measured by battle deaths, and the frequency of war. These findings were later confirmed in more detailed studies^6–13^. The power-law distribution regarding war size, characterized as fat-tailed, implies the possible occurrence of *black swan* events, such as World War I (WWI) or World War II (WWII). The severity of other forms of human violence, such as civil war, insurgency, or terrorist attacks, has also been shown to follow the power law^6,14–16^. Finding statistical patterns in the severity of war and other human violence has inspired the exploration of the mechanism for the escalation of violence, typically attributed to ‘critical phenomena’ resulting from the operation of positive feedback loops^6,7,15–20^. This robust statistical pattern can also be used to infer the actual number of casualties of inadequately recorded wars or to examine the risk of the future occurrence of enormous wars such as WWI or WWII^8,9,11,13,21^.

While many of the previous studies have examined spatial features, such as the size of war, in their exploration of statistical patterns, this study focuses on temporal features, such as the timing of conflict occurrence, because temporal features are more fundamentally related to decision-making by states regarding military action, which is the core process of conflict occurrence. Furthermore, robust statistical patterns in the temporal features can be used more directly to predict the future occurrence of conflicts because prediction is a task along the temporal dimension. In fact, as the prediction of event occurrences, such as war, alliance formation, and revolutions, is at the heart of international relations theory, scholars in the discipline have realized that a proper arrangement of time is required to achieve sophisticated predictions^22,23^. Whether we should explicitly theorize the relationship between time and the events under study^23,24^ or not^25^, it is now standard practice to account for time in modelling the occurrence of events by including splines or polynomials^22,24^ or by using Cox duration models^26,27^. These attempts suggest that time plays a vital role in international events^26^, the most notable of which is war. Therefore, exploring the temporal structures of conflict processes is a prerequisite to understanding and predicting wars.

In this study, we show that power law, which is well documented in the spatial aspects of conflicts, such as the size of war, also holds in their temporal aspects. Previous studies examining the timing of conflict occurrence^11,28,29^, which are few compared to those examining the size of war, suggest that the timing fails to follow power law. We argue that power law was unobserved in these studies because they looked at the timing of all conflicts rather than distinguishing dyads in disputes. To distinguish dyads, we propose to use the interconflict interval (ICI), the interval between the end of a conflict in a dyad and the start of the subsequent conflict in the same dyad. Using rigorous statistical tests, we confirmed that ICI samples compiled from the history of interstate conflicts followed a power-law distribution. To account for the empirically observed properties of ICIs, we then built a hypothetical model that incorporates the supposed nature of interstate conflicts. Finally, we evaluated the prediction of this model by further elaborating the temporal structure of the ICI distributions.

## Results

### Terminology: interstate war, militarized interstate dispute, and armed conflict

First, we specify the definitions of interstate wars, militarized interstate disputes, and armed conflicts. Precise definitions of the first two terms are provided by the Correlates of War (COW) Project (https://correlatesofwar.org/). An *interstate war* is a series of sustained battles between the armed forces of two or more states that have resulted in at least 1,000 battle deaths^30^. A *militarized interstate dispute* is defined as a set of incidents involving the deliberate, overt, government-sanctioned, and government-directed threat, display, or use of force between two (or more) states^30,31^. Interstate wars are a subset of militarized interstate disputes. Therefore, a quotient set of militarized interstate disputes by interstate wars is a set of militarized interstate disputes *short of* wars.

In a militarized interstate dispute, military action taken by one or both states is preceded by political issues, such as conflicting national interests or disagreements over foreign policy. The inclusion of both interstate wars and militarized interstate disputes short of wars in our analysis is appropriate if we stand on the view that the use of force, in any form, should be a way to resolve international issues, whereas previous studies on the severity of militarized interstate disputes have focused only on interstate wars^7,9,11^. Our view resonates with the famous thesis of Prussian general and war philosopher Carl von Clausewitz^32^:

“War is merely the continuation of policy by other means” (Clausewitz 1832^33^)

He derived a corollary from this thesis, arguing that:

“The political object—the original motive of the war—will thus determine both the military objective to be reached and the amount of effort it requires.” (Clausewitz 1832^33^)

The use of force in an actual war must be proportional to the political objectives. Thus, Clausewitz’s arguments motivated us to address militarized interstate disputes short of wars and interstate wars without distinction. In this paper, militarized interstate dispute will be concisely referred to as *armed conflict* or just *conflict*.

### Dataset of interstate conflict

We used Dyadic MID Version 4.02 (MID 4.02), a dataset provided by the COW Project^30^. The dataset records armed conflicts between 1816 and 2014. Each conflict is specified by a dyad (a pair of states) engaged in the conflict and the start and end dates of the conflict.

### Interconflict interval

**Figure 1:**
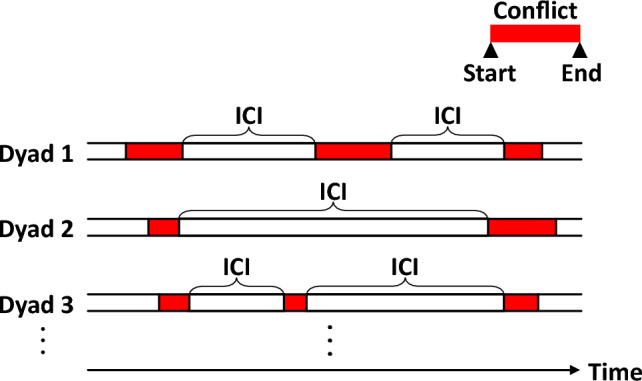
Interconflict intervals (ICIs). The ICI is the interval between the end of a conflict in a dyad and the start of the next conflict in the same dyad. Each conflict is indicated by the red rectangle.

We sought to identify robust statistical patterns behind the temporal structure of the occurrence of interstate conflicts. The interconflict interval (ICI) is the critical quantity to this end and is defined as the interval between the end of a conflict in a dyad and the start of the next conflict in the same dyad (Fig. 1). We obtained 2,369 ICI samples from MID 4.02, each measured in days. These ICI samples were collected from all dyads.

### Testing the power-law hypothesis of ICIs

**Figure 2:**
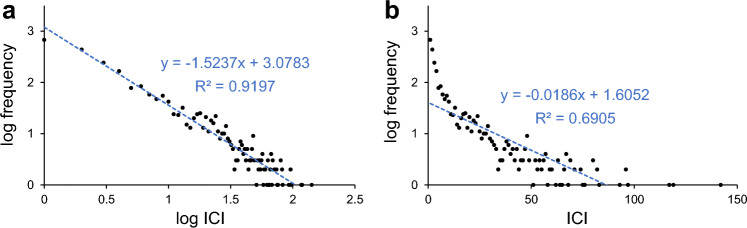
Distribution of 2,369 ICI samples collected from all dyads is shown in log–log (**a**) and linear-log (**b**) plots. The bin width for the distribution was chosen as 365.25 days (~ one year). The dashed blue line in each panel indicates the linear regression results.

First, the distribution of these 2369 ICIs binned with a width of 365.25 days (~ one year) was examined in log–log and linear-log plots. Falling into a straight line in a log–log or linear-log plot is characteristic of a power-law or exponential distribution, respectively. The linear regression results in both plots suggested that the ICIs followed a power-law distribution (${R}^{2}=0.9179$, Fig. 2a) instead of an exponential distribution (${R}^{2}=0.6905$, Fig. 2b).

We also examined the power-law fitting using a more elaborate statistical test proposed by Clauset et al. (2009)^34^, which we call the Clauset-Shalizi-Neuman (CSN) test. The empirical distributions of the ICI samples would have an artificial upper bound because of the limitations of the recording period, even if they were generable from power-law distributions with infinitely extended tales (see Methods for further details). To consider the possible existence of artificial upper bounds, we used a modified version of the CSN test (mCSN test).

The power-law hypothesis to be examined using the mCSN test is mathematically expressed as follows: $p\left(x\right)={x}^{-\gamma }/Z\left(\gamma \right)$ (${x}_{{\text{min}}}\le x\le {x}_{{\text{max}}}$). Here, the argument $x$ takes an integer value; $\gamma$ is the exponent of the power-law distribution; ${x}_{{\text{min}}}$ and ${x}_{{\text{max}}}$ are the lower and upper bounds of the range where the power law holds, respectively; and $Z\left(\gamma \right)={\sum }_{x={x}_{{\text{min}}}}^{{x}_{{\text{max}}}}{x}^{-\gamma }$ is the normalization constant equal to the partition function. The details of the mCSN test are provided in the Methods. In brief, we first estimated the exponent $\gamma$ and lower bound ${x}_{{\text{min}}}$ and then calculated the $p$-value. Let $\widehat{\gamma }$ and ${\widehat{x}}_{{\text{min}}}$ be the estimated values of $\gamma$ and ${x}_{{\text{min}}}$, respectively. The upper bound ${x}_{{\text{max}}}$ was used as the control parameter. Therefore, $\widehat{\gamma }$ and ${\widehat{x}}_{{\text{min}}}$ as well as the $p$-value were given as a function of ${x}_{{\text{max}}}$. Clauset et al. (2009)^34^ proposed conservative decision criteria: If $p\le 0.1$, the power-law hypothesis is ruled out; otherwise, it is plausible. The same criteria were used in this study.

**Figure 3:**
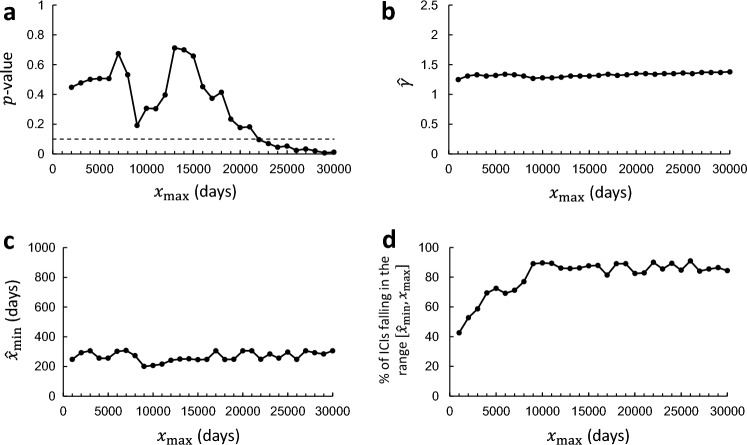
Results of the mCSN test applied to 2,369 ICI samples collected from all dyads. This test reveals the plausibility of the power-law hypothesis expressed in the form: $p\left(x\right)\propto {x}^{-\gamma }$ for ${x}_{{\text{min}}}\le x\le {x}_{{\text{max}}}$, where ${x}_{{\text{min}}}$ and ${x}_{{\text{max}}}$ are the lower and upper bounds of the domain in which the power law holds, respectively. The upper bound ${x}_{{\text{max}}}$ is treated as a control parameter, and the optimal values of $\gamma$ and ${x}_{{\text{min}}}$ are estimated for each value of ${x}_{{\text{max}}}$. (**a**) The $p$-value of the mCSN test is plotted as a function of ${x}_{{\text{max}}}$. The horizontal dashed line indicates the criteria of 0.1, for the $p$-value above which the power-law hypothesis is plausible. (**b**) The estimated power-law exponent $\widehat{\gamma }$ is plotted as a function of ${x}_{{\text{max}}}$. (**c**) The estimated lower bound ${\widehat{x}}_{{\text{min}}}$ is plotted as a function of ${x}_{{\text{max}}}$. (**d**) The ratio of ICIs (out of the total, 2369) that fall in the power-law holding domain (${\widehat{x}}_{{\text{min}}}\le x\le {x}_{{\text{max}}}$) is plotted as a function of ${x}_{{\text{max}}}$.

The results of the mCSN tests are shown in Fig. 3. The $p$-value exceeded the criteria of 0.1 (indicated by the horizontal dashed line in Fig. 3a) for up to ${x}_{{\text{max}}}$ slightly longer than 20,000 days (~ 55 years) (Fig. 3a). In Fig. 3b,c, we observe that $\widehat{\gamma }$ and ${\widehat{x}}_{{\text{min}}}$ are almost constant with ${x}_{{\text{max}}}$; $\widehat{\gamma }$ is approximately 1.3 and ${\widehat{x}}_{{\text{min}}}$ is approximately 250 days ($<$ 1 year). From these observations, we conclude that the ICI obeys the power law for the range of 250–20,000 days. Approximately 80% of the ICI samples were within this range for ${x}_{{\text{max}}}=\mathrm{20,000}$ (Fig. 3d).

### Information-theoretic model of interstate conflict

Next, we built a hypothetical model that accounted for the observed power-law properties of ICIs. Consider a dyad of states A and B. Suppose that the $n$-th conflict ${C}_{n}$ is provoked by either state. Following the terminology of the COW project^30^, we refer to the state that triggers conflict as the initiator and the opponent state as the target. Conflict ${C}_{n}$ is characterized by the time of its occurrence and the military actions taken during the conflict. Let this time and the military actions be represented by stochastic variables ${T}_{n}$ and ${\mathbf{X}}_{n}$, respectively. For simplicity, we assume that the period bounded by the start and end of a conflict contracts to a point. Therefore, ${T}_{n}$ takes the real value ${t}_{n}\in {R}^{1}$. In contrast, corresponding to the various possibilities of the course of a war, ${\mathbf{X}}_{n}$ would take multidimensional values ${\mathbf{x}}_{n}$ that would be categorical or numerical. Furthermore, each military action may be led by either the initiator or the target, as contingent switching between offence and defence is the case during the course of war. Nevertheless, in the following discussion, we formally address ${\mathbf{X}}_{n}$ without addressing its mathematical details.

After the settlement of conflict ${C}_{n}$, a postconflict order is established, whether or not it is what the initiator desires. Then, either state, which is discontent with the status of this order and wants to change it to what is more favourable to it, intends to provoke the next conflict ${C}_{n+1}$. The initiator of conflict ${C}_{n+1}$ may or may not be the same as that of conflict ${C}_{n}$. The time of conflict ${C}_{n+1}$ and military actions taken during this conflict are represented by the stochastic variables ${T}_{n+1}$ and ${\mathbf{X}}_{n+1}$, respectively.

**Figure 4:**
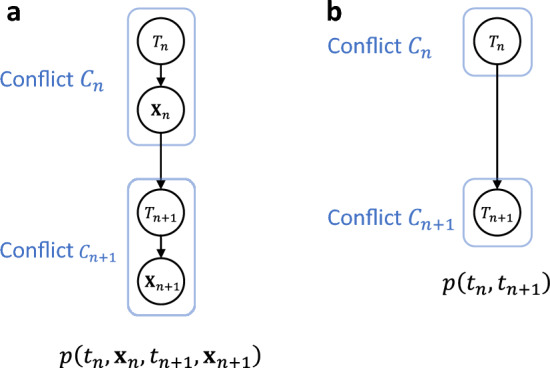
Graphical models describing the causal relations between stochastic variables representing consecutive occurrences of conflicts ${C}_{n}$ and ${C}_{n+1}$. Stochastic variables ${T}_{n}$ and ${\mathbf{X}}_{n}$ represent the time of occurrence of conflict ${C}_{n}$ and the military operations taken during the course of this conflict, respectively. (**a**) A graphical model representing the causal relations between ${T}_{n}$, ${\mathbf{X}}_{n}$, ${T}_{n+1}$, and ${\mathbf{X}}_{n+1}$, corresponding to the joint probability $p\left({t}_{n}, {\mathbf{x}}_{n}, {t}_{n+1}, {\mathbf{x}}_{n+1}\right)$. The amount of information transferred from $\left\{{T}_{n},{\mathbf{X}}_{n}\right\}$ to $\left\{{T}_{n+1},{\mathbf{X}}_{n+1}\right\}$ is equivalent to the amount of information mutually exchanged between the two states through their engagement in consecutive conflicts ${C}_{n}$ and ${C}_{n+1}$. (**b**) A graphical model representing the causal relation between ${T}_{n}$ and ${T}_{n+1}$, obtained by marginalizing the graphical model in (**a**) over ${\mathbf{X}}_{n}$ and ${\mathbf{X}}_{n+1}$ and corresponding to the probability $p\left({t}_{n}, {t}_{n+1}\right)$.

The end (purpose) of war is to attain a political objective, and military action is a means to achieve this objective. Both the initiator and the target conceived their own purposes. For instance, the initiator’s purpose is to compel the other to submit to its will, whereas the target’s purpose is to compel the initiator to withdraw. The variable ${T}_{n}$ describes when a political disagreement between the two states becomes critical and either or both states decide to resolve this by force. In this respect, ${T}_{n}$ reflects the purpose of the war. In fact, ${T}_{n}$ encodes when the purpose is conceived but does not say what it is. Because the means should be aligned with the purpose, ${\mathbf{X}}_{n}$ instead of ${T}_{n}$ reflect what the purpose is. As conceiving a purpose precedes choosing the means, ${T}_{n}$ causally precedes ${\mathbf{X}}_{n}$. In summary, the causal relationships between ${T}_{n}$, ${\mathbf{X}}_{n}$, ${T}_{n+1}$, and ${\mathbf{X}}_{n+1}$ are expressed by the graphical model shown in Fig. 4a, which corresponds to the joint probability $p\left({t}_{n}, {\mathbf{x}}_{n}, {t}_{n+1}, {\mathbf{x}}_{n+1}\right)$.

According to information theory, the amount of information carried by stochastic variables $\left\{{T}_{n},{\mathbf{X}}_{n}\right\}$ is measured by entropy:

1 $$H[T_{n} ,\;{\mathbf{X}}_{n} ] = - \int {dt_{n} d{\mathbf{x}}_{n} \;p\left( {t_{n} ,\;{\mathbf{x}}_{n} } \right)\log p\left( {t_{n} ,\;{\mathbf{x}}_{n} } \right)} .$$

The amount of information successfully received by the stochastic variables $\left\{{T}_{n+1},{\mathbf{X}}_{n+1}\right\}$ out of the total sent by $\left\{{T}_{n},{\mathbf{X}}_{n}\right\}$, which is precisely the entropy $H\left[{{T}_{n}, \mathbf{X}}_{n}\right]$, is measured by mutual information:

2 $$\begin{gathered} I\left[ {\left\{ {T_{n} ,\;{\mathbf{X}}_{n} } \right\},\;\left\{ {T_{n + 1} ,\;{\mathbf{X}}_{n + 1} } \right\}} \right] \hfill \\ = \int_{{}} {dt_{n} d{\mathbf{x}}_{n} dt_{n + 1} d{\mathbf{x}}_{n + 1} p\left( {\left\{ {t_{n} ,\;{\mathbf{x}}_{n} } \right\},\;\left\{ {t_{n + 1} ,\;{\mathbf{x}}_{n + 1} } \right\}} \right)\log \frac{{p\left( {\left\{ {t_{n} ,\;{\mathbf{x}}_{n} } \right\},\;\left\{ {t_{n + 1} ,\;{\mathbf{x}}_{n + 1} } \right\}} \right)}}{{p\left( {t_{n} ,\;{\mathbf{x}}_{n} } \right)p\left( {t_{n + 1} ,\;{\mathbf{x}}_{n + 1} } \right)}}} \;. \hfill \\ \end{gathered}$$

It is reasonable to hypothesize that the amount of information transferred from $\left\{{T}_{n},{\mathbf{X}}_{n}\right\}$ to $\left\{{T}_{n+1},{\mathbf{X}}_{n+1}\right\}$ corresponds to the amount of information mutually exchanged between the two states through their engagement in consecutive conflicts ${C}_{n}$ and ${C}_{n+1}$.

Equation (2) can be arranged as

3 $$I\left[ {\left\{ {T_{n} ,\;{\mathbf{X}}_{n} } \right\},\;\left\{ {T_{n + 1} ,\;{\mathbf{X}}_{n + 1} } \right\}} \right] = I\left[ {T_{n} ,\;T_{n + 1} } \right] + I\left[ {{\mathbf{X}}_{n} ,\;{\mathbf{X}}_{n + 1} |T_{n} ,\;T_{n + 1} } \right],$$

where

$$\begin{gathered} I\left[ {T_{n} ,\;T_{n + 1} } \right] = H\left[ {T_{n} } \right] + H\left[ {T_{n + 1} } \right] - H\left[ {T_{n} ,\;T_{n + 1} } \right]\;, \hfill \\ I\left[ {{\mathbf{X}}_{n} ,\;{\mathbf{X}}_{n + 1} |T_{n} ,\;T_{n + 1} } \right] = H\left[ {{\mathbf{X}}_{n} |T_{n} } \right] + H\left[ {{\mathbf{X}}_{n + 1} |T_{n + 1} } \right] - H\left[ {{\mathbf{X}}_{n} ,\;{\mathbf{X}}_{n + 1} |T_{n} ,\;T_{n + 1} } \right]\;. \hfill \\ \end{gathered}$$

Thus, the total amount of information exchanged between the two states through their engagement in consecutive conflicts ${C}_{n}$ and ${C}_{n+1}$ is equivalent to the sum of the mutual information $I\left[{T}_{n}, {T}_{n+1}\right]$ and $I\left[{\mathbf{X}}_{n},{\mathbf{X}}_{n+1}|{T}_{n}, {T}_{n+1}\right]$. We interpret $I\left[{T}_{n}, {T}_{n+1}\right]$ as the amount of information exchanged at the national strategy level and $I\left[{\mathbf{X}}_{n},{\mathbf{X}}_{n+1}|{T}_{n}, {T}_{n+1}\right]$ as that exchanged at the military operation level. The latter is particularly relevant to the extent to which battle lessons from military operations taken during conflict ${C}_{n}$ influence those taken during conflict ${C}_{n+1}$. We considered conflicts to be event units, and the success or failure of military operations conducted during each conflict was outside the scope of this study. That is, our main interest is the communication between the two states at the national strategy level. Therefore, we focus on $I\left[{T}_{n}, {T}_{n+1}\right]$. In doing so, we marginalize the graphical model in Fig. 4a over ${\mathbf{X}}_{n}$ and ${\mathbf{X}}_{n+1}$ to obtain the graphical model in Fig. 4b, which corresponds to the probability $p\left({t}_{n},{t}_{n+1}\right)=\int d{\mathbf{x}}_{n}d{\mathbf{x}}_{n+1}p\left({t}_{n}, {\mathbf{x}}_{n}, {t}_{n+1}, {\mathbf{x}}_{n+1}\right)$.

Thus, our consideration leads to the intriguing notion that the amount of information exchanged between the two states at the national strategy level depends only on the relative timing of their engagement in consecutive conflicts. In the present study, we followed this notion without further verification. Future studies should address historical cases of interstate conflicts to verify this notion empirically.

The interval between conflicts ${C}_{n}$ and ${C}_{n+1}$, now given by ${T}_{n+1}-{T}_{n}$, also served as a stochastic variable. Once $p\left({t}_{n}, {t}_{n+1}\right)$, the joint probability of ${T}_{n}$ and ${T}_{n+1}$, is known, and $p\left({t}_{n+1}-{t}_{n}\right)$, the distribution of ${T}_{n+1}-{T}_{n}$, can be easily calculated. Therefore, we determined the functional forms of $p\left({t}_{n}, {t}_{n+1}\right)$. Information theory states that a probability distribution that exists maximizes entropy. In general, entropy maximization is performed under constraints that specify the objects or phenomena of interest. To define the constraints in our case, we assume that states A and B, struggling with their national interests and survival, will behave according to the trade-off between the principle of promptness and the principle of seriousness.

The need for the first principle of promptness can be easily understood. Suppose that the status quo is unfavourable for state A. The longer this status continues, the more state A will incur losses in the national interest. To prevent further losses, state A intends to take military action in any form against state B to change the status quo as promptly as possible. The principle of promptness implies a behavioural tendency to avoid wasting time.

The second principle, seriousness, implies a deeper understanding of ‘coordinated violence’, which is referred to as the characteristics of war at the beginning of this paper. Remind Clausewitz’s fundamental thesis: “War is merely the continuation of policy by other means.” We now interpret this thesis from the perspective of modern information theory, rephrasing it as follows: “The use of military force is a means of interstate communication.” To formulate interstate communication through force in the framework of information theory, it is useful to note Clausewitz’s argument.

“War is no pastime; it is no mere joy in daring and winning, no place for irresponsible enthusiasts. It is a serious means to a serious end” (Clausewitz 1832 ^33^).

This implies that the state responds seriously to an opponent’s move. (Serious responses do not necessarily mean rational responses; see Discussion). In other words, the rivalling states use force not haphazardly but in a coordinated manner trying to make the communication between them as efficient as possible. In our mathematical formulation, therefore, variables $\left\{{T}_{n},{\mathbf{X}}_{n}\right\}$ and $\left\{{T}_{n+1},{\mathbf{X}}_{n+1}\right\}$ (Fig. 4a) should as mutually dependent as possible. Even after marginalization (Fig. 4b), the same should go for the remaining variables ${T}_{n}$ and ${T}_{n+1}$. The principle of seriousness also implies that there is no room for behavioural redundancy in the theatre, where rivalling states act per their national interests and survival.

To achieve ‘a serious end’ with ‘a serious means,’ the principle of promptness alone is inadequate. Suppose that conflict occurs at a high frequency, following this principle; however, the timing of each conflict occurrence is statistically independent of that before it (this is the case if a conflict occurs following a Poisson process). This implies that conflict occurs only erratically, which is the opposite of seriousness.

The constraints for entropy maximization to determine the functional forms of $p\left({t}_{n}, {t}_{n+1}\right)$ are defined by the principles above. For mathematical simplicity, we consider the case where ${T}_{n}$ and ${T}_{n+1}$ take continuous values: $-\infty <{t}_{n}<x+\Delta \le {t}_{n+1}<+\infty$, where $\Delta \left(>0\right)$ is the minimum length of ICI. The constraint representing the principle of promptness is defined as the force required to reduce ${T}_{n+1}-{T}_{n}$. Because ${T}_{n+1}-{T}_{n}$ is a stochastic variable, its statistical mean is reduced, not its raw value. There are several types of statistical means, such as arithmetic or geometric means. Therefore, the following question arises: What kind of statistical means should we choose? More specifically, what kinds of statistical means of ${T}_{n+1}-{T}_{n}$ do states behave to reduce? We leave aside this problem and instead consider the generalized mean, which can express a variety of statistical means by varying the parameterization. We later demonstrate that the parameterization is determined by the second principle.

The generalized mean of ${T}_{n+1}-{T}_{n}$ is given by

4 $${\text{E}}_{{T_{n} ,\;T_{n + 1} }}^{{({\text{gen}})}} \left[ {T_{n + 1} - T_{n} } \right] = \left( {\int\limits_{ - \infty }^{ + \infty } {dt_{n} \int\limits_{{t_{n} + \Delta }}^{ + \infty } {dt_{n + 1} } } \;p\left( {t_{n} ,\;t_{n + 1} } \right)\left( {t_{n + 1} - t_{n} } \right)^{m} } \right)^{1/m} ,$$

where $m$ is the parameter characterizing the generalized mean and $\Delta \left(>0\right)$ is the minimum length of the possible interval between conflicts ${C}_{n}$ and ${C}_{n+1}$. By varying $m$, Eq. (4) yields various statistical methods. For example, Eq. (4) is equal to the arithmetic mean for $m=1$ and approaches the geometric mean for $m\to 0$.

The joint entropy of ${T}_{n+1}$ and ${T}_{n}$ is hence given by

5 $$\begin{gathered} S\left[ {T_{n} ,\;T_{n + 1} } \right] = - \int\limits_{ - \infty }^{ + \infty } {dt_{n} \int\limits_{{t_{n} + \Delta }}^{ + \infty } {dt_{n + 1} \;} } p\left( {t_{n} ,\;t_{n + 1} } \right)\log p\left( {t_{n} ,\;t_{n + 1} } \right) \hfill \\ - \gamma \log {\text{E}}_{{T_{n} ,\;T_{n + 1} }}^{{({\text{gen}})}} \left[ {T_{n + 1} - T_{n} } \right] - \lambda \left( {\int\limits_{ - \infty }^{ + \infty } {dt_{n} \int\limits_{{t_{n} + \Delta }}^{ + \infty } {dt_{n + 1} } } \;p\left( {t_{n} ,\;t_{n + 1} } \right) - 1} \right)\;. \hfill \\ \end{gathered}$$

The first term on the right-hand side represents Shannon’s entropy. The second term is introduced according to the first principle of promptness and expresses the force required to reduce the generalized mean of ${T}_{n+1}-{T}_{n}$; the coefficient $\gamma \left(>0\right)$ controls the strength of this force. The third term, where $\lambda$ is a Lagrange multiplier, ensures the normalization condition that $p\left({t}_{n}, {t}_{n+1}\right)$ are summed to unity. Maximizing entropy (5) with respect to $p\left({t}_{n}, {t}_{n+1}\right)$ and rescaling $\gamma /{{\text{E}}}_{{T}_{n+1}, {T}_{n}}^{({\text{gen}})}\left[{T}_{n+1}, {T}_{n}\right]\to \gamma$ yields

6 $$p\left( {t_{n} ,\;t_{n + 1} } \right) = \frac{1}{{Z\left( {\gamma ,\;m} \right)}}\exp \left[ { - \frac{\gamma }{m}\left( {t_{n + 1} - t_{n} } \right)^{m} } \right],$$

where

7 $$Z\left( {\gamma ,\;m} \right) = \int\limits_{ - \infty }^{ + \infty } {dt_{n} \int\limits_{{t_{n} + \Delta }}^{ + \infty } {dt_{n + 1} \;} } \exp \left[ { - \frac{\gamma }{m}\left( {t_{n + 1} - t_{n} } \right)^{m} } \right]$$

is the normalization factor. As expected, Eq. (6) becomes equal to the exponential distribution $p\left({t}_{n}, {t}_{n+1}\right)\propto {\text{exp}}\left[-\gamma \left({t}_{n+1}-{t}_{n}\right)\right]$ for $m=1$ and approaches the power-law distribution $p\left({t}_{n}, {t}_{n+1}\right)\propto {\left({t}_{n+1}-{t}_{n}\right)}^{-\gamma }$ for $m\to 0$^35^. For $p\left({t}_{n}, {t}_{n+1}\right)$ to be normalized, $m$ should be nonzero positive.

**Figure 5:**
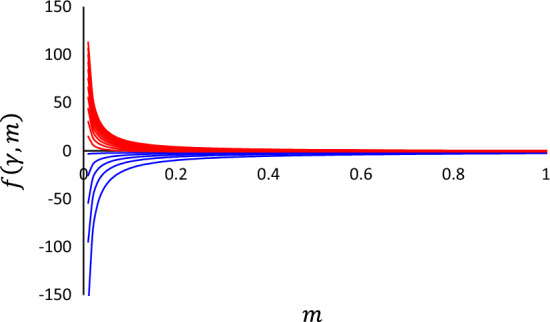
Mutual information $I\left[{T}_{n},{T}_{n+1}\right]=f\left(\gamma , m\right)+{\text{constant}}$ as functions of $\gamma$ and $m$. The analytical form of $f\left(\gamma , m\right)$ is given by Eq. (10). The curves in the coordinate plane plot $f\left(\gamma , m\right)$ as a function of $m$ for different values of $\gamma$ (varied from 0.2 to 3.0 in 0.2 increments). Curves for $\gamma <1$ and $\gamma \ge 1$ are colored blue and red, respectively. For any value of $\gamma \ge 1$, $f\left(\gamma , m\right)$ is maximally extremized at $m\to +0$.

Next, we demonstrate that the value of $m$ is determined by the second principle of seriousness. As previously discussed, this principle makes stochastic variables ${T}_{n}$ and ${T}_{n+1}$ mutually dependent as much as possible. Information theory states that the mutual dependence between stochastic variables can be estimated by mutual information:

8 $$I\left[ {T_{n} ,\;T_{n + 1} } \right] = \int\limits_{ - \infty }^{ + \infty } {dt_{n} \int\limits_{{t_{n} + \Delta }}^{ + \infty } {dt_{n + 1} } } \;p\left( {t_{n} ,\;t_{n + 1} } \right)\log \frac{{p\left( {t_{n} ,\;t_{n + 1} } \right)}}{{p\left( {t_{n} } \right)p\left( {t_{n + 1} } \right)}},$$

where $p\left({t}_{n}\right)={\int }_{-\infty }^{+\infty }p\left({t}_{n}, {t}_{n+1}\right)d{t}_{n+1}$ and $p\left({t}_{n+1}\right)={\int }_{-\infty }^{+\infty }p\left({t}_{n}, {t}_{n+1}\right)d{t}_{n}$ are marginal probabilities. Using the forms of Eqs. (6) and (7) and taking $\Delta \to 0$, we can analytically calculate the right-hand side of Eq. (8) to obtain

9 $$I\left[ {T_{n} ,\;T_{n + 1} } \right] = f\left( {m,\;\gamma } \right) + {\text{constant,}}$$

10 $$f\left( {m,\;\gamma } \right) = \frac{1}{m}\log \gamma - \left( {\frac{1}{m} - 1} \right)\log m - \log \Gamma \left( \frac{1}{m} \right) - \frac{1}{m},$$

where $\Gamma \left(\cdot \right)$ denotes the gamma function. The principle of seriousness argues that mutual information $I\left[{T}_{n}, {T}_{n+1}\right]$ should be maximized. Figure 5 shows $f\left(m, \gamma \right)$ as functions of $m \left(>0\right)$ and $\gamma \left(>0\right)$. For each value of $\gamma \ge 1$, $f\left(m, \gamma \right)$ is maximized for $m\to +0$ (Fig. 5, red curves). Thus, the principle of seriousness, which is embodied by the maximization of mutual information, leads to the power-law distribution of $\tau \equiv {t}_{n+1}-{t}_{n}$:

11 $$p\left( \tau \right) = \frac{\gamma - 1}{{\Delta^{ - \gamma + 1} }}\tau^{ - \tau } \quad \left( {\Delta \le \tau < + \infty } \right)$$

with $$\gamma \ge 1$$.

### Dilution of the power-law process: relation between the model and observation

**Figure 6:**
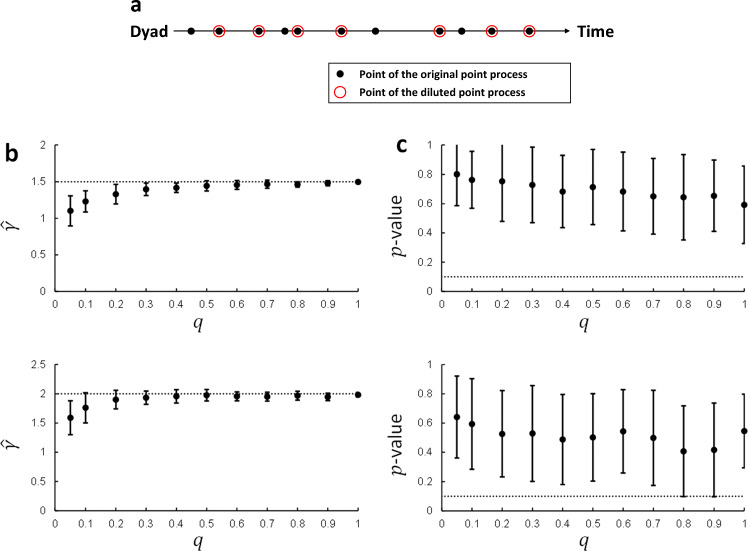
(**a**) Illustration of a point process whose point-to-point intervals are supposed to follow a power-law distribution. The chain of filled black circles represents an original point process supposed to follow a power-law distribution. This process is diluted by probabilistically maintaining or discarding each point. The chain of maintained points, indicated by the blank red circles surrounding them, constitutes a diluted point process. The original point process models true occurrences of conflict in the history, regardless of whether they are recorded in the dataset. The diluted point process models conflict occurrences that are actually recorded in the dataset. (**b**) An original point process following a power-law distribution ($p\left(x\right)\propto {x}^{-\gamma }$ for $x=1, 2, 3, \cdots$) is diluted with the maintaining probability $q$ (hence with the discarding probability $1-q$). We conducted the mCSN test applied to the point-to-point intervals of 100 processes obtained by the probabilistic dilution. The power-law exponent $\widehat{\gamma }$ estimated by this test is plotted as a function of $q$ (upper and lower panels for $\gamma =1.5$ and $\gamma =2.0$, respectively). The horizontal dotted lines in both panels indicate the power-law exponent $\gamma$ of the original process. (**c**) The $p$-value of the mCSN test is plotted as a function of $q$ (upper and lower panels for $\gamma =1.5$ and $\gamma =2.0$, respectively). The horizontal dotted lines in both panels indicate the criteria of 0.1, for the $p$-value above which the power-law hypothesis is plausible. In (**b**) and (**c**), the error bars indicate the standard deviations.

Because the interval between consecutive occurrences of conflict, but not the duration of each conflict itself, was of interest, we prescribed the duration of each conflict to be contracted to a point in time. With this mathematical simplification, the conflict occurrence in each dyad can be viewed as a point process (Fig. 6a, filled black circles). Our information-theoretic model predicts that the point-to-point intervals of this process follow a power-law distribution.

However, we should be aware of the possibility of a recording bias. Some cases of militarized interstate disputes may have been overlooked in the collection of data and were not recorded. Therefore, the point process compiled from the dataset is obtained by diluting the original point process generated by the model (Fig. 6, blank red circles).

Therefore, it is necessary to examine whether the diluted point process also follows the power law and, if so, whether the power-law exponent for the diluted point process is equal to that for the original point process. The power-law distribution given by Eq. (11) has a lower bound $\Delta \left(>0\right)$ in the domain. For $\Delta \to 0$, the diluted point process follows the same power law as the original process, owing to the scale-invariant property of the power-law distribution. However, in reality, $\Delta$ would be slightly greater than zero because a minimum length of time is required (for example, to redeploy resources) before the invocation of the next conflict. To examine whether the point process obtained by diluting the original power-law process with $\Delta >0$ also follows the power law, we conducted the following numerical experiments: A sample of the point process is generated so that point-to-point intervals follow a power-law distribution with $\Delta =1$. The generated point process is then diluted with probability $q$; that is, each point is left and abandoned with probabilities $q$ and $1-q$, respectively. The point-to-point intervals collected from the dilution process then undergo the mCSN test to calculate the $p$-value and estimate the fitted power-law exponent $\widehat{\gamma }$.

The experimental results are shown in Fig. 6b,c. The $p$-value (Fig. 6c) and fitted power-law exponent $\widehat{\gamma }$ (Fig. 6b) are plotted as a function of the probability $q$. For any $q$, the $p$-value averaged over 100 calculations is substantially larger than the criterion of 0.1 (Fig. 6c), indicating that the power-law hypothesis for the diluted point process is plausible. The fitted power-law exponent decreases from the original value for $\gamma$ as $q$ decreases (Fig. 6b). These results demonstrate that point-to-point intervals collected from the dilution process, which model observed ICIs, also follow a power-law distribution, although its exponent is reduced from the original $\gamma$.

### Mixture of power-law distributions

Our information-theoretic model predicts that conflict occurrences in each dyad follow the power law. In addition, the power-law exponent may differ for each dyad because it originates from a predefined value for $\gamma$, which is not necessarily consistent for every dyad. We will later see that the power-law exponent inferred from the real data varies from dyad to dyad. Therefore, the distribution of ICIs collected from all dyads, which we have shown to follow a power-law distribution (Fig. 2), should be a mixture of power-law distributions originating from different dyads, with different exponents. Therefore, verifying whether a mixture of power-law distributions can be approximated accurately using a single power-law distribution is necessary. Indeed, we have accomplished this, the detailed descriptions of which are provided in the Methods.

### Testing the power-law hypothesis in individual dyads

Our information-theoretic model predicts that ICIs collected from individual dyads will follow separate power laws. To test this prediction, we applied the mCSN test to seven dyads: CHN-RUS, CHN-US, GMY-FRN, IND-PAK, IRN-IRQ, ISR-SYR, and RUS‒US. We used the following abbreviations: CHN (China), FRN (France), GMY (Germany), IND (India), IRN (Iran), IRQ (Iraq), ISR (Israel), SYR (Syria), and US (the United States). We chose these seven dyads because they were politically relevant and provided the number $N\ge 20$ of ICI samples that were likely eligible for statistical examination.

The results of the mCSN tests are presented in Table 1. In this test, ${x}_{{\text{max}}}$ was chosen as $\underset{n}{{\text{max}}}{x}_{n}$, which was the maximum ICI sample for each dyad. Noticeably, the power-law hypothesis of ICIs was plausible for all the dyads we examined ($p>0.1$ for every dyad). Our model argues that the power-law exponent is not less than 1.0. Although the obtained power-law exponent for GMY-FRN is 0.96, this value is very close to 1.0 and is not considered seriously contradicting the argument. The ratio ${N}_{{\text{D}}}/N$, where $N$ and ${N}_{{\text{D}}}$ are the total number of ICIs and the number of ICIs equal to or larger than the estimated lower bound ${\widehat{x}}_{{\text{min}}}$, respectively, was substantially large (> 0.7) for every dyad, indicating that the power law holds for a wide range of ICIs (Table 1).

**Table 1:** Results of the mCSN test of the power-law hypothesis expressed in the form: $p\left(x\right)={x}^{-\gamma }/Z\left(\gamma \right)$ for ${x}_{{\text{min}}}\le x\le {x}_{{\text{max}}}$. Here, the value of ${x}_{{\text{max}}}$ is chosen as the maximum length of ICI samples, and the normalization factor is given by $Z\left(\gamma \right)={\sum }_{{x=x}_{{\text{min}}}}^{{x}_{{\text{max}}}}{x}^{-\gamma }$. $N$: the number of ICI samples for each dyad. ${\widehat{x}}_{{\text{min}}}$: the estimated value of ${x}_{{\text{min}}}$. ${N}_{{\text{D}}}$: the number of ICI samples within domain ${\widehat{x}}_{{\text{min}}}\le x\le {x}_{{\text{max}}}$. ${N}_{{\text{D}}}/N$: the ratio of ICI samples within the domain. $\widehat{\gamma }$: the estimated value of the power-law exponent $\gamma$. The bottom row lists the $p$-value of the mCSN test. For the $p$-value larger than the criteria of 0.1, as indicated by the asterisk (*), the power-law hypothesis is plausible.

	CHN-RUS	CHN-US	FRN-GMY	IND-PAK	IRN-IRQ	ISR-SYR	RUS‒US
$N$	38	29	20	33	29	33	39
${\widehat{x}}_{{\text{min}}}$	155	196	46	219	104	153	278
${N}_{{\text{D}}}$	34	26	20	24	27	25	28
${N}_{{\text{D}}}/N$	0.895	0.897	1.000	0.727	0.931	0.758	0.718
$\widehat{\gamma }$	1.11	1.51	0.96	2.17	1.65	1.56	2.01
$p$-value	0.8852*	0.2151*	0.9656*	0.2114*	0.7105*	0.9438*	0.293*

We also compared the power-law hypothesis with the alternative hypothesis that the ICIs follow an exponential distribution. From a set of ICI samples such that ${\widehat{x}}_{{\text{min}}}\le {\text{ICI}}\le {x}_{{\text{max}}}$, 100 pseudo datasets were synthesized using the bootstrap process. We calculated the maximum log-likelihood of the exponential and power-law distributions for each synthesized dataset. A paired $t$-test was conducted to examine whether the maximum log-likelihood of the power-law distribution (${\text{log}}{L}^{({\text{p}}.{\text{l}}.)}$) was significantly larger than that of the exponential distribution (${\text{log}}{L}^{({\text{exp}})}$). The results summarized in Table 2 show that the power-law distribution is significantly more plausible than the exponential distribution for every dyad. Thus, we concluded that the ICIs in each dyad followed a power-law distribution, which is consistent with the predictions of our model.

**Table 2:** The upper row lists the mean difference ${\langle {\text{log}}{\widehat{L}}^{\left({\text{p}}.{\text{l}}.\right)}\rangle }_{B}-{\langle {\text{log}}{\widehat{L}}^{\left({\text{exp}}\right)}\rangle }_{B}$ for each dyad. The mean ${\langle {\text{log}}{\widehat{L}}^{\left({\text{p}}.{\text{l}}.\right)}\rangle }_{B}$ was calculated by averaging the loglikelihood for the power-law hypothesis over $B=100$ pseudoseries of ICIs generated using the bootstrap process. The mean ${\langle {\text{log}}{\widehat{L}}^{\left({\text{exp}}\right)}\rangle }_{B}$ of the loglikelihood for the exponential-distribution hypothesis was calculated similarly. Positive values of the quantity ${\langle {\text{log}}{\widehat{L}}^{\left({\text{p}}.{\text{l}}.\right)}\rangle }_{B}-{\langle {\text{log}}{\widehat{L}}^{\left({\text{exp}}\right)}\rangle }_{B}$ indicate that the power-law hypothesis is more likely than the exponential-distribution hypothesis. The bottom row lists the $p$-value of the paired $t$-test for each dyad to demonstrate the significance of the positivity or negativity of this quantity.

	CHN-RUS	CHN-US	FRN-GMY	IND-PAK	IRN-IRQ	ISR-SYR	RUS‒US
${\langle {\text{log}}{\widehat{L}}^{\left({\text{p}}.{\text{l}}.\right)}\rangle }_{B}-{\langle {\text{log}}{\widehat{L}}^{\left({\text{exp}}\right)}\rangle }_{B}$	1.425	12.5185	5.507	1.944	7.284	3.148	9.358
$p$-value	4.90E-07	3.65E-56	3.88E-28	8.26E-08	1.50E-14	2.04E-27	5.24E-26

**Figure 7:**
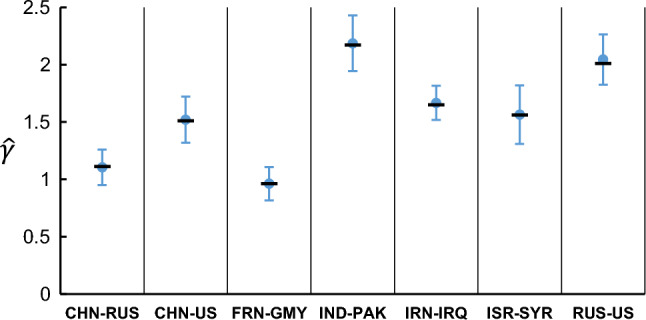
The power-law exponent varies from dyad to dyad. The estimated power-law exponent $\widehat{\gamma }$ for each dyad is indicated by the filled black bar. To confirm the stability of this estimation, 100 pseudoseries of ICIs were synthesized using the bootstrap process, for each of which the power-law exponent was re-estimated. The filled blue circle and error bar indicate the mean and standard deviation of $\widehat{\gamma }$ calculated using the bootstrap process.

The estimated power-law exponent $\widehat{\gamma }$ varies from dyad to dyad, ranging from ~ 1.0 to ~ 2.0 (Table 1). These estimated values were robust, as confirmed by bootstrap analysis (Fig. 7). Variable $\widehat{\gamma }$ across dyads, albeit robustly estimated in each dyad, supports the notion that the distribution of total ICIs, which has been shown to obey the power law with an exponent of ~ 1.3 (Fig. 2), is a mixture of power-law distributions with variable exponents.

The mCSN test ensures that the power-law hypothesis is more plausible than the exponential-distribution hypothesis in the estimated domain ${\widehat{x}}_{{\text{min}}}\le x\le {x}_{{\text{max}}}$. However, this does not necessarily exclude the possibility that the exponential-distribution hypothesis is more plausible than the power-law hypothesis in another domain. This may have occurred, especially when the number of ICIs was small, as in the present case. To examine this possibility, we conduct an mCSN test to examine the exponential-distribution hypothesis. The results are summarized in Table S1. In contrast to the mCSN test of the power-law hypothesis, which gives a $p$-value larger than the criteria of 0.1 for any of the seven dyads, the $p$-value of the mCSN test of the exponential-distribution hypothesis is below the criteria for three dyads (CHN-US, IRN-IRQ, and RUS‒US). Therefore, the exponential distribution hypothesis for the estimated domains was excluded from these dyads. The mean difference ${\langle {\text{log}}{L}^{\left({\text{p}}.{\text{l}}.\right)}\rangle }_{B}-{\langle {\text{log}}{\widehat{L}}^{\left({\text{exp}}\right)}\rangle }_{B}$ calculated using the bootstrap process was positive for two dyads (CHN-US and FRN-GMY) (Table S2), implying that the power-law hypothesis is more likely than the exponential distribution hypothesis in the estimated domains for these dyads. Furthermore, the variability in the estimated $\widehat{\lambda }$ appeared to be more sprawling across the dyads (Fig. S1) than the estimated $\widehat{\gamma }$ (Fig. 7), which implies a less robust estimation of $\widehat{\lambda }$. Although it is difficult to judge which hypothesis is more plausible, comparing the results shown in Tables 1, 2, and Fig. 7 with those shown in Tables S1, S2, and Fig. S1 strongly suggests that fitting a power-law distribution to the ICI samples for each dyad is more suitable.

A log-normal distribution can mimic a power-law distribution for a relatively large interval. A log-normal distribution may fit the data better than a pure power-law distribution. To reject this possibility, we compared the power-law hypothesis with the hypothesis that ICIs follow a log-normal distribution. For this, we used Akaike’s information criterion (AIC) to consider the difference in the number of parameters; a power-law distribution is characterized by a single parameter, whereas a log-normal distribution is characterized by two parameters. The AIC was significantly lower for power-law distributions than for log-normal distributions (Table S8). Thus, the power-law distribution is more likely than the log-normal distribution.

### ICIs are independent and identically distributed

**Figure 8:**
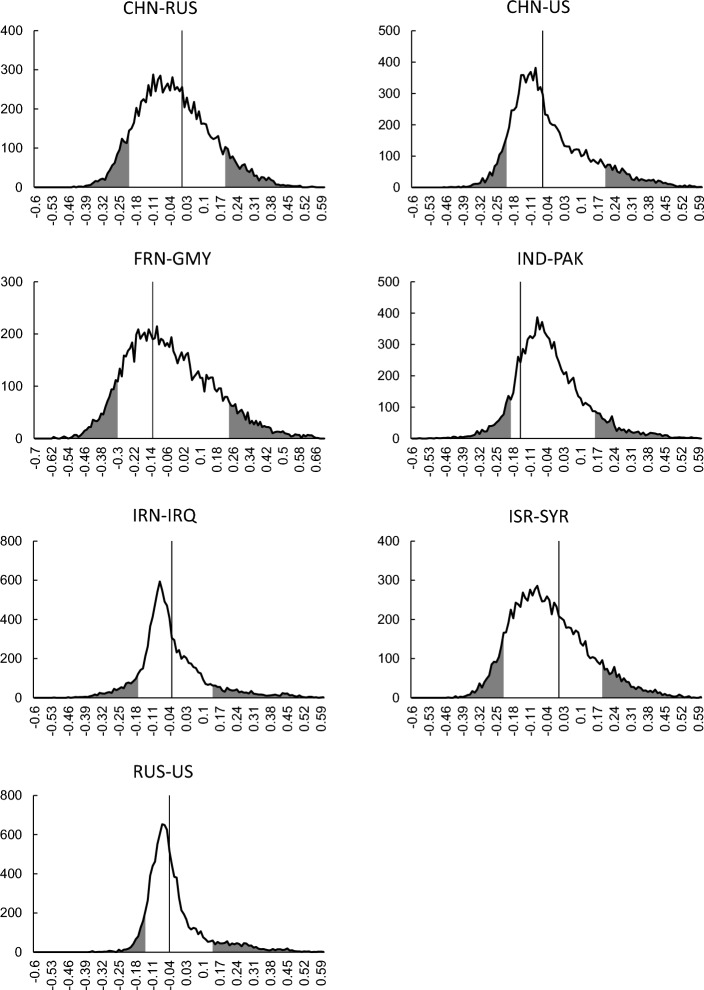
ICIs in each dyad are independently generated from an identical distribution. From the actual series of ICIs in each dyad, 10,000 pseudoseries were synthesized by bootstrapping. The ICIs in each pseudoseries conform to independent generation from an identical distribution. The curve in each panel shows the distribution of first-order autocorrelations calculated for the 10,000 pseudoseries. The leftward and rightward 10% areas (shaded grey) reject the null hypothesis that the actual series of ICIs fails to exhibit nonvanishing first-order autocorrelation. The vertical solid line in each panel indicates the first-order autocorrelation ${a}^{(1)}\left({\varvec{\uptau}}\right)$ of the actual series.

Our information-theoretic model predicts that the ICIs in each dyad are generated independently from an identical power-law distribution. In contrast, the interval ${\tau }_{n}$ between the timing of the $\left(n-1\right)$-th and $n$-th fatal attacks in insurgency and terrorism approximately follows a power-law progress curve ${\tau }_{n}={\tau }_{1}{n}^{-b}$, most typically with escalation ($b>0$) and sometimes with de-escalation ($b<0$)^16,18^. We conducted the following statistical experiment to confirm that the actual generation of ICIs in each dyad was independent and identically distributed and that the observed power-law distribution of ICIs was due to neither escalation nor de-escalation. Let ${\tau }_{n}$ be the $n$-th ICI generated in a certain dyad and ${a}^{(1)}\left({\varvec{\uptau}}\right)$ be the first-order autocorrelation calculated for the ICI series ${\varvec{\uptau}}=\left\{{\tau }_{1},\cdots ,{\tau }_{N}\right\}$ (see Methods for details). If series ${\varvec{\uptau}}$ followed escalation or de-escalation, ${a}^{(1)}\left({\varvec{\uptau}}\right)$ would be significantly high. From this series, $B=\mathrm{10,000}$ pseudo series were synthesized by bootstrapping. These pseudo series follow independent and identically distributed processes. We then calculated the distribution of the first-order autocorrelations over these pseudoseries. For this distribution, which normally has a single peak around zero, a rejection area is defined rightward with a significance level ${p}_{s}$, for which we chose a conservative value (${p}_{s}=0.1$). This rejection area (the rightward area shaded gray in each panel of Fig. 8) corresponds to the possibility that the positive correlation between ${\tau }_{n}$ and ${\tau }_{n+1}$ is significantly high, as is the case for escalation and de-escalation. Another rejection area was defined to the left at the same significance level (${p}_{s}=0.1$). This area (the leftward area shaded gray in each panel of Fig. 8), for which the negative correlation between ${\tau }_{n}$ and ${\tau }_{n+1}$ is significantly high, indicates the tendency that a longer ICI is followed by a shorter ICI, and vice versa, thereby producing oscillatory progress.

We can test the null hypothesis that series ${\varvec{\tau}}$ fails to have nontrivial (i.e., significantly positive or negative) first-order autocorrelation by examining the location of the value for ${a}^{(1)}\left({\varvec{\uptau}}\right)$ in the distribution; if it enters either of the rejection areas, the null hypothesis is ruled out. For any of the seven dyads (CHN-RUS, CHN-US, GMY-FRN, IND-PAK, IRN-IRQ, ISR-SYR, and RUS‒US), the value for ${a}^{(1)}\left({\varvec{\uptau}}\right)$, indicated by the vertical lines in Fig. 8, is outside the rejection areas. Therefore, the null hypothesis is not rejected for these dyads. It is unlikely that a series without a first-order autocorrelation will have a higher-order autocorrelation. Thus, we concluded that the actual generation of ICIs in each dyad follows an independent and identically distributed process, which is consistent with the predictions of our model.

## Discussion

War is an extreme form of collective human behaviour characterized by coordinated violence. According to an inductive approach, we have shown that this nature of war is substantiated by the temporal patterns of conflict occurrence that obey power law. The critical quantity for exploring temporal patterns is the interconflict interval (ICI) (Fig. 1). Using rigorous statistical tests, we found that ICI samples compiled from the history of interstate conflicts from 1816 to 2014 followed a power-law distribution (Figs. 2 and 3). To account for this empirical observation, we propose a hypothetical model built on the fundamental thesis on war, which was raised by Clausewitz two hundred years ago and is here reinterpreted from the perspective of modern information theory: “The use of force is a means of interstate communication”, which is in accordance with coordinated violence, the particular nature of war. The model predicts that a series of ICIs in each dyad are independently generated from an identical power-law distribution. We statistically analyzed individual dyads separately and obtained results consistent with the predictions of the model (Tables 1 and 2; Fig. 8).

This study reveals for the first time that the interevent time distributions obey the power law in conflicts between regular states, while Picoli et al. (2014)^36^ suggested the power-law properties of interevent time distributions in insurgency.

To test the power law hypothesis of ICIs collected from all dyads or collected separately from individual dyads, we used a modified version of the rigorous statistical method proposed by^34^. This method, which we call the mCSN test, calculates the $p$-value and then judges whether a specific hypothesis (e.g., the power-law hypothesis or the exponential-distribution hypothesis) of ICIs is plausible if the obtained $p$-value is larger than the criteria of $0.1$; otherwise, it is ruled out. Noticeably, the $p$-value for ICIs collected from all dyads, plotted as a function of ${x}_{{\text{max}}}$ (Fig. 3a), shows a conspicuous trough at approximately 9000 days (~ 25 years), even though the $p$-value around this trough is slightly larger than 0.1, indicating that the power-law hypothesis is barely plausible. We suppose that this trough was attributable to the interwar period bounded by the end of WWI (1918) and the beginning of WWII (1939). The power-law hypothesis of ICIs premises that the conflict process in each dyad is independent of those in other dyads. However, this premise was violated during WWI and WWII when many countries became involved in war almost simultaneously and automatically, according to either side of the opposing camps they had taken. The resulting excess number of ICIs, whose lengths were comparable to those of the interwar period, eventually caused a substantial deviation in the tail shape of the distribution from the power law. To confirm this supposition, we removed ICIs related to either of the world wars by leaving ICIs whose end and start dates were before the start of 1914 and after the end of 1945, respectively, and then applied the mCSN to the remaining samples. With this prescription, trough levels disappeared (Supplementary Materials, Fig. S2). This implies that WWI and WWII, in which countries all over the world entered a state of war simultaneously due to alliance relationships, were historically unique events.

Except during WWI and WWII, the results obtained in the present study support the idea that conflict processes in individual dyads are independent of each other. Nevertheless, examining in more detail the influence of conflict processes in some dyads on others, if any, is of substantial interest, as recent studies suggest that higher-order interactions, as well as pairwise (i.e., first-order) interactions between states, affect conflict occurrence^37–39^.

The ICI samples collected from all dyads were well suited to a power-law distribution with an exponent of ~ 1.3 (Fig. 3b). In general, the value of the power-law exponent is related to the frequency of event occurrence; the larger the power-law exponent is, the more frequent the events. As the dataset includes interstate conflicts over the past 200 years (1816–2014), a question arises: Is the power-law exponent consistent or changing over the past 200 years? A recent study conducting out-of-sample cross-validation demonstrated that causal models of war vary periodically^40^. To address this, we also examined the power-law hypothesis by dividing the entire period (1816–2014) into the following eras: (i) the first half of the nineteenth century (1816–1858); (ii) the second half of the nineteenth century (1859–1899); (iii) the first half of the twentieth century lasting from 1900 to just after WWII (1946); (iv) the Cold War era (1947–1989), and (v) the post-Cold War era (1990 ~ the present (2014)). The results obtained demonstrate that as time passes, the value of the power-law exponent gradually increases ($\widehat{\gamma }=$ 1.18, 1.0, 1.22, 1.7, and 1.71 for eras (i), (ii), (iii), (iv), and (v), respectively; see Supplementary Materials, Table S3, and Fig. S3). A gradual increase in the frequency of conflict over the last 200 years is the most naïve interpretation of these observations. However, the observed increase in the power-law exponent can be attributed to a recording bias. Some interstate conflicts, especially those in older eras, may have been overlooked when compiling the data.

The $p$-value for the second era (iii), lasting from 1900 to 1946, was $0.0286 \left(<0.1\right)$ (Table S3). Therefore, the power-law hypothesis is not plausible. As demonstrated in Fig. S2, the ruling out of the power law hypothesis was most likely caused by the inclusion of the interwar period in this era. Therefore, we trimmed the last six years of this era. ICIs compiled from this trimmed-off era, lasting from 1900 to 1938, no longer involved an excess number of ICIs compared to the interwar period. Indeed, we obtained $p=0.449 \left(>0.1\right)$ for this trimmed-off era, say (iii’), confirming the plausibility of the power-law hypothesis (Table S3).

This study demonstrates that the ICI, the interval bounded by consecutive conflicts occurring in the same dyad, follows the power law. In contrast, Richardson’s earlier works^28,29^ suggested that the timing of onset of full-scale wars (interstate wars, in our terminology), occurring anywhere in the world, obeys a Poisson process; that is, the interval between the timing of onset of consecutive wars occurring anywhere in the world follows an exponential distribution. Therefore, we sought to examine whether the timing of onset of interstate conflicts, counted without specifying the dyad, also follows an exponential distribution. To this end, we defined the dyad-unconditioned interconflict interval (DUC-ICI, Fig. S4). Considering the possibility that the rate of conflict occurrence may change over the years^11^, we used the above division of the entire period into five eras. DUC-ICI samples were compiled separately for each era and then underwent the mCSN tests. The results of the mCSN tests for the power-law hypothesis (Fig. S5, Tables S4, and S5) and the exponential-distribution hypothesis (Fig. S6, Tables S6, and S7) indicate that the DUC-ICIs for each era are more likely to follow an exponential distribution than a power-law distribution, consistent with Richardson’s suggestion.

We do not ask whether the action taken by either state is a rational means of achieving its political objective, whether the political objective itself is reasonable, or whether it is achieved as intended by settling the conflict. This contrasts with the game-theoretical approach to interstate wars, which assumes that actors behave rationally. This approach has been favoured in mainstream international relations theory. For instance, in his game-theoretic model with the assumption that states are ‘rational’ actors, James Fearon (1995)^2^ demonstrated that ‘inefficient’ (in the sense that they cannot reach a deal that is mutually less costly than an armed confrontation) war can take place between them due to a lack of communication, intentional or unintentional. Our information-theoretic model, which regards armed violence as a means of communication in and of itself, may appear contradictory to Fearon's model, as we argue that serious acts do not necessarily conform to rational ones. Nevertheless, we also argue that states only consider the timing of the previous conflict in deciding when to initiate an armed conflict, thereby disregarding its means and costs. Therefore, our insights resonate with the motivation behind the Fearon model.

Our information-theoretic model argues that ICIs are independently generated from an identical power-law distribution in each dyad. The absence of a first-order autocorrelation for a series of actual ICIs in the individual dyads supports this notion. However, the present study did not examine whether autocorrelation resides in the size of interstate conflicts; for instance, every time a conflict occurs, its size grows or shrinks. This issue will be addressed in future studies.

Our model is based on an information-theoretic formulation of the hypothesis that military force is a form of interstate communication. The lines of evidence obtained by the statistical analysis of the MID 4.02 dataset support the plausibility of this hypothesis. This hypothesis might contradict the widespread view that interstate war arises from a lack of communication between states. However, from an information-theoretic perspective, the observed power-law properties of ICI are the hallmark of maximally efficient communication through violent means.

Power laws are ubiquitously observed in the time course of human behaviour, such as e-mail/surface-mail correspondence and web browsing^41,42^. What makes our findings unique lies in the argument that the power-law properties of ICI arise from the interaction between rival states, which we model as a form of communication in the information-theoretic framework. Here, we draw on Clausewitz’s statement, which supports this argument.

“War, however, is not the action of a living force upon a lifeless mass … but always the collision of two living forces.” (Clausewitz 1823 ^33^).

Indeed, two classes of models, priority queuing models and modulated Markov processes, have been discussed to account for the power-law properties of interevent intervals empirically observed in human behaviour. In contrast to our information-theoretic model, these models assume that individuals behave independently. Priority queuing models^41–44^ assume that a person has a prioritized list of tasks and executes any task at a time that is probabilistically selected from this list according to this priority. The waiting time of a task from its entry into the list to its execution follows the power law (of exponent 1.0 or 1.5). Each individual creates a prioritized list of tasks that are independent of others. For the modulated Markov process^45–48^, the power law is accounted for as a consequence of the combination of Poisson processes, which model the behaviour of every individual as the sporadic execution of tasks with circadian or weekly cycles. Thus, modulated Markov process models lack the perspective of the interaction between living agents. The above overview of priority queuing and modulated Markov process models suggests that these models cannot explain the observed power-law properties of ICIs, which are thought to be an essential consequence of the interaction between rivalling states. The interactions between rival agents are at the heart of our information theory model. This implies that our information-theoretic model is more favourable than priority-queuing models or modulatory Markov processes for accounting for the power-law properties of ICIs.

A relevant example can be found in a completely different field of neuroscience, where interspike intervals (ISIs) in neuronal spike trains have been observed to follow a power law^49,50^. Computational neuroscientists examined the power-law properties of ISIs using the principles of information theory^50^. Neurons, like states, are communicators, and information processing in the brain is the totality of the communication between neurons. Power laws may be a hallmark of communication between real-world actors, such as states or neurons.

This study focuses on armed conflicts between normal states. This contrasts with the recent trend in the discipline, which is devoted to asymmetric warfare, such as insurgency or terrorism, rather than armed conflicts between normal states^6,14–16,18,36^. The September 11th attacks might have triggered this trend. However, the full-scale war in Ukraine, started by the Russian invasion on February 24, 2022, disenchanted us from the illusion that armed conflict between regular states may be outdated^51^.

## Methods

### Dataset

This study used Dyadic MID Data 4.02 (MID 4.02), the dataset that can be downloaded from a public repository run by the COW Project (https://correlatesofwar.org). The dataset records militarized interstate disputes (MIDs) from 1816 to 2014. Each MID in the dataset is specified with a dyad (a pair of states) engaged in this MID, the start and end days of this MID, and values for other covariates. For instance, the covariate ${\text{WAR}}$ takes $1$ for interstate war and $0$ for short-of-war MID.

The dataset MID 4.02 (dyadic_mid_4.02.csv, downloadable from https://correlatesofwar.org/data-sets/mids/) includes 3,544 MIDs (see dyadic_mid_ICI.csv stored at https://github.com/HO299792458/PowerLawICI). There appear 931 dyads in the dataset (see dyad_name_list.csv, dyad_id_list.csv and double_counted_dyads.csv at the same place). These dyads experienced at least one MID in the history. Out of them, 449 (~ 48%) dyads experienced MID only once, and the remaining 482 (~ 52%) dyads experienced MIDs twice or more (see dyad_ mid.csv and mid_ dyad_freq.csv at the same place).

### Interconflict interval (ICI)

The interconflict interval (ICI) is defined as the interval between a conflict (MID) in a dyad and the start of the next conflict in the same dyad (Fig. 1). Let ${t}_{c}^{\left({\text{start}}\right)}$ and ${t}_{c}^{\left({\text{end}}\right)}$ be the start and end times (dates) of the $c$-th conflict that occurred in a certain dyad, respectively. ICIs were collected from this dyad by calculating ${t}_{c}^{\left({\text{start}}\right)}-{t}_{c-1}^{\left({\text{end}}\right)}$ for $c\ge 1$. If ${t}_{c}^{({\text{start}})}-{t}_{c-1}^{\left({\text{end}}\right)}\le 0$, the $c-1$-th and $c$-th conflicts are regarded as being continued, and therefore, nonpositive ICIs are excluded from sampling. Because ${t}_{0}^{\left({\text{start}}\right)}$ and ${t}_{0}^{\left({\text{end}}\right)}$ are not applicable (NA), ${t}_{1}^{\left({\text{start}}\right)}-{t}_{0}^{\left({\text{end}}\right)}$ is also not applicable (NA).

To sample ICIs from the dataset, we first created a data file that lists $\left({t}_{c-1}^{\left({\text{start}}\right)},{t}_{c-1}^{\left({\text{end}}\right)},{t}_{c}^{\left({\text{start}}\right)},{t}_{c}^{\left({\text{end}}\right)}\right)$ for every dyad (see dyadic_mid_ICI.csv stored in https://github.com/HO299792458/PowerLawICI). Deleting NAs and nonpositive values, we finally obtained 2,369 well-defined ICIs (see the same data file). Note that 449 (~ 48%) dyads having experienced only one MID provided no ICI samples.

Sampling ICIs separately from seven politically relevant dyads, CHN-RUS, CHN-US, GMY-FRN, IND-PAK, IRN-IRQ, ISR-SYR, and RUS‒US, were performed in the same manner. For each of these dyads, we created a data file that lists $\left({t}_{c-1}^{\left({\text{start}}\right)},{t}_{c-1}^{\left({\text{end}}\right)},{t}_{c}^{\left({\text{start}}\right)},{t}_{c}^{\left({\text{end}}\right)}\right)$ in order of ${t}_{c-1}^{\left({\text{end}}\right)}$ (see < DYAD_NAME > _ici.csv at https://github.com/HO299792458/PowerLawICI)). Deleting NA and nonpositive values, we created a file that lists well-defined ICIs in order of their generation in the history (namely, a series of ICIs). In doing so, we obtained 38, 29, 20, 33, 29, 33, and 39 ICIs for CHN-RUS, CHN-US, GMY-FRN, IND-PAK, IRN-IRQ, ISR-SYR, and RUS‒US, respectively.

### Goodness-of-fit test for the power-law hypothesis

Clauset et al. (2009)^34^ proposed a goodness-of-fit test to examine whether a given set of samples $\left\{{x}_{1},\cdots , {x}_{N}\right\}$ follows a power-law distribution. This test, which we call the Clause-Shalizi-Newman (CSN) test, was designed to examine the power-law properties of spatial features, such as war size, earthquake magnitude, and urban population. Samples of spatial features, if they follow power-law distributions, include a number of large-sized events because the long tails characterizing power-law distributions imply the likely occurrence of large-sized events. Therefore, the power-law hypothesis to be examined by the original CSN test is mathematically expressed as $p\left(x\right)={x}^{-\gamma }/\zeta \left(\gamma , {x}_{{\text{min}}}\right)$ $\left({x}_{{\text{min}}}\le x\right)$, where ${\sum }_{{x}_{{\text{min}}}}^{+\infty }{x}^{-\gamma }=\zeta \left(\gamma , {x}_{{\text{min}}}\right)$ is the generalized zeta function. Note that the domain ${x}_{{\text{min}}}\le x$ has no upper bound.

In contrast, caution is required when applying the CSN test to temporal features such as ICIs. Sampling ICIs from the dataset MID 4.02 is restricted by the recording period used to construct this dataset, which is approximately 200 years, from 1816 to 2014. Therefore, even if ICIs were generable from power-law distributions without upper bounds, the lengths of the ICI samples collected from MID 4.02 would never exceed the recording period. This means that the empirical distribution of ICIs has an upper bound, above which no sample exists. Furthermore, individual dyads had their own ages, some of which were much shorter than the recording period. For instance, the Russia-Ukraine dyad was approximately 23 years old in 2014 (the final year of the recording period). The length of the ICI samples collected from the dyads never exceeded their age. Consequently, the empirical distribution of the ICIs collected from all dyads would have an effective upper bound that might be much smaller than the recording period, above which the power-law distribution would no longer fit the data well.

Therefore, to fit a power-law distribution to temporal features, such as ICIs, we must consider the upper bound ${x}_{{\text{max}}}$ in addition to the lower bound ${x}_{{\text{min}}}$. The power law implies the likely occurrence of long-term events for temporal features. However, such long-term events could not be recorded because the recording period was limited. In contrast, when recording spatial features, large-sized events, such as enormous wars (WWI or WWII), enormous earthquakes, or megacities, would never be overlooked.

To examine the power-law hypothesis of the ICIs, the original CSN test should be modified by considering the possible presence of upper bounds. The procedure for the modified CSN (mCSN) test, used to examine the power-law hypothesis for ICIs in the present study, was as follows: Let $p\left(x\right)$ be the probability distribution of variable $X$. We consider the case where $x$ takes discrete values measured in days. The power-law hypothesis to be examined by the mCSN is mathematically expressed in the following form: $p\left(x\right)={x}^{-\gamma }/Z\left(\gamma \right)$, where $\gamma$ is the power-law exponent and $Z\left(\gamma \right)={\sum }_{x={x}_{{\text{min}}}}^{{\text{max}}}{x}^{-\gamma }$ is the normalization factor equivalent to the partition function. Let $\mathcal{D}=\left\{{x}_{1}, \cdots , {x}_{N}\right\}$ be the data. Samples that are smaller than ${x}_{{\text{min}}}$ or larger than ${x}_{{\text{max}}}$, if they exist, are excluded from $\mathcal{D}$ because we want to test the hypothesis in the domain ${x}_{{\text{min}}}\le x\le {x}_{{\text{max}}}$. The log-likelihood is then given as

12 $$\log L\left( \gamma \right) = \sum\limits_{n = 1}^{N} {\log p\left( {x_{n} } \right)} = - \gamma \sum\limits_{n = 1}^{N} {\log x_{n} } - N\log Z\left( \gamma \right).$$

The value of the power exponent $\gamma$ is determined using the maximum likelihood estimate (MLE). The estimation can be performed by direct numerical maximization of $\log L\left(\gamma \right)$. The model fitted by MLE is denoted as $\mathcal{M}$.

The distance between data $\mathcal{D}$ and the hypothesis is measured by the Kolmogorov‒Smirnov (KS) statistic ${D}_{{\text{KS}}}$ defined by

13 $$D_{{{\text{KS}}}} = \mathop {\max }\limits_{{x_{{{\text{min}}}} \le x \le x_{{{\text{max}}}} }} \left| {S\left( x \right) - P\left( x \right)} \right|,$$

where $S\left(x\right)=\left(\mathrm{the number of }{x}_{n}\ge x\right)/N$ is the cumulative distribution function (CDF) for the empirical data. $P\left(x\right)={\sum }_{x\ge {x}^{\prime}}p\left({x}^{\prime}\right)$ is the CDF for the fitted model $\mathcal{M}$.

A large number $S$ of power-law distributed data, ${\mathcal{D}}_{1}, \cdots , {\mathcal{D}}_{S}$, are synthesized from $\mathcal{M}$. Each data point has the same number $N$ of elements as the empirical data $\mathcal{D}$. We fit each synthetic data ${\mathcal{D}}_{s}$ to its own power-law model ${\mathcal{M}}_{s}.$ Then, we calculate the KS statistics ${D}_{s}$ for ${\mathcal{D}}_{s}$ relative to ${\mathcal{M}}_{s}.$ Then, we count the fraction of time that ${D}_{s}$ is larger than $D$, which serves as the $p$-value of this test. Clauset, Shalizi, and Newman (2009) set the conservative decision criteria for the test: If $p\le 0.1$, the power-law hypothesis for the data $\mathcal{D}$ is ruled out; otherwise, it is plausible. We conducted a goodness-of-fit test for the ICI samples with $S =10, 000$ times the generation of synthetic data.

### Mixture of power-law distributions well approximated by a single power-law distribution

We prove that the likelihood of a mixture of power-law distributions is as close as possible to that of a single power-law distribution. Although the proof is not mathematically rigorous, it provides an intuitive understanding of why a mixture of power-law distributions can be approximated using a single power-law distribution in several cases.

Consider a mixture of power-law distributions:

14 $$p\left( x \right) = \sum\limits_{k = 1}^{K} {\pi \left( k \right)p\left( {x|k} \right)} ,$$

where $p\left(x|k\right)$ is the power-law distribution with exponent ${\gamma }_{k} \left(>1\right)$,

15 $$p\left( {x|k} \right) = \frac{{\gamma_{k} - 1}}{{x_{{{\text{min}}}} }}x^{{ - \gamma_{k} }} \quad \left( {x \ge x_{{{\text{min}}}} } \right).$$

We assume that the domain of each component distribution has a lower bound ${x}_{{\text{min}}}$ but infinitely extends rightward without an upper bound. The loglikelihood of the data $\mathcal{D}=\left\{{x}_{1}, \cdots , {x}_{N}\right\}$ is

16 $$\log L_{{{\text{mix}}}} = \sum\limits_{n = 1}^{N} {\log \left( {\sum\limits_{k = 1}^{K} {\pi \left( k \right)\frac{{\gamma_{k} - 1}}{{x_{{{\text{min}}}}^{{ - \gamma_{k} + 1}} }}x_{n}^{{ - \gamma_{k} }} } } \right)} .$$

Using Jensen’s inequality, one can arrange this as

17 $$\log L_{{{\text{mix}}}} \ge \sum\limits_{n = 1}^{N} {\sum\limits_{k = 1}^{K} {\pi \left( k \right)\log \left( {\frac{{\gamma_{k} - 1}}{{x_{{{\text{min}}}}^{{ - \gamma_{k} + 1}} }}x_{n}^{{ - \gamma_{k} }} } \right)} } = \sum\limits_{n = 1}^{N} {\left[ {\log \frac{1}{{x_{{{\text{min}}}}^{ - \gamma + 1} }}x_{n}^{ - \gamma } + \sum\limits_{k = 1}^{K} {\pi \left( k \right)\log \left( {\gamma_{k} - 1} \right)} } \right]} \;,$$

where $\gamma \equiv {\sum }_{k=1}^{K}\pi \left(k\right){\gamma }_{k}$. The right-hand side is hence denoted by

18 $$Q_{{{\text{mix}}}} \equiv \sum\limits_{n = 1}^{N} {\left[ {\log \frac{1}{{x_{{{\text{min}}}}^{ - \gamma + 1} }}x_{n}^{ - \gamma } + \sum\limits_{k = 1}^{K} {\pi \left( k \right)\log \left( {\gamma_{k} - 1} \right)} } \right]} .$$

The log-likelihood for a single power-law distribution of the exponent $\gamma$ is given as follows:

19 $$\begin{gathered} \log L_{{{\text{single}}}} = \sum\limits_{n = 1}^{N} {\log \left( {\frac{\gamma - 1}{{x_{{{\text{min}}}}^{ - \gamma + 1} }}x_{n}^{ - \gamma } } \right)} \hfill \\ = \sum\limits_{n = 1}^{N} {\left[ {\log \frac{1}{{x_{{{\text{min}}}}^{ - \gamma + 1} }}x_{n}^{ - \gamma } + \log \left( {\sum\limits_{k = 1}^{K} {\pi \left( k \right)\left( {\gamma_{k} - 1} \right)} } \right)} \right]} \hfill \\ \ge \sum\limits_{n = 1}^{N} {\left[ {\log \frac{1}{{x_{{{\text{min}}}}^{ - \gamma + 1} }}x_{n}^{ - \gamma } + \sum\limits_{k = 1}^{K} {\pi \left( k \right)\log \left( {\gamma_{k} - 1} \right)} } \right]} = Q_{{{\text{mix}}}} \;. \hfill \\ \end{gathered}$$

Jensen’s inequality was used to derive the inequality in the third row of Eq. (19). According to probabilistic machine-learning theories^52^, we can solve $\pi \left(k\right)$ and ${\gamma }_{k}$ by maximizing ${Q}_{{\text{mix}}}$. For ${\text{log}}{L}_{{\text{single}}}\ge {Q}_{{\text{mix}}}$, an increase in ${Q}_{{\text{mix}}}$ leads to an increase in ${\text{log}}{L}_{{\text{single}}}$. Therefore, maximizing ${Q}_{{\text{mix}}}$ causes the single power-law distribution with $\gamma ={\sum }_{k=1}^{K}\pi \left(k\right){\gamma }_{k}$ to fit the data more closely. If ${\text{log}}{L}_{{\text{single}}}\ge {\text{log}}{L}_{{\text{mix}}}$, then the single power-law distribution inherently fits the data better than the mixture. Now, consider the case where ${\text{log}}{L}_{{\text{single}}}<{\text{log}}{L}_{{\text{mix}}}$. Since ${\text{log}}{L}_{{\text{mix}}}>{\text{log}}{L}_{{\text{single}}}>{Q}_{{\text{mix}}}$, the mixture fits the data better than a single power-law distribution. Nevertheless, as ${Q}_{{\text{mix}}}$ becomes as close to ${\text{log}}{L}_{{\text{mix}}}$ as possible by its maximization, ${\text{log}}{L}_{{\text{single}}}$, which lies between them, approaches ${\text{log}}{L}_{{\text{mix}}}$. This implies that the mixture can be approximated using a single power-law distribution.

### Testing the power-law hypothesis of ICIs in a single dyad

The set of 2369 ICIs collected from all dyads, for which the power-law hypothesis was examined using the mCSN test, was a collection of subsets of ICIs collected from individual dyads. Our information-theoretic model predicts that the power-law hypothesis holds for individual dyads. To test this prediction, we examined whether the ICIs collected from a single dyad followed a power-law distribution. For this purpose, ${x}_{{\text{max}}}$ is set to the maximum ICI.

We examined seven politically relevant dyads (CHN-RUS, CHN-US, GMY-FRN, IND-PAK, IRN-IRQ, ISR-SYR, and RUS‒US dyads), each of which provided the number of ICIs eligible for statistical analysis. Nevertheless, the number was relatively low (from 20 to 39 ICIs), which may have caused an overestimation of the $p$-value of the CSN test. Therefore, an obtained $p$-value larger than 0.1, which implies the plausibility of the power-law hypothesis, does not necessarily mean that competing hypotheses, typically the exponential distribution hypothesis, are ruled out. To confirm that the power law hypothesis is more likely than the exponential distribution hypothesis, we compared the log-likelihood between the power law and exponential distribution hypotheses.

Let ${\varvec{\uptau}}=\left\{{\tau }_{1}, \cdots , {\tau }_{N}\right\}$ be the set of ICIs collected from a certain dyad, where ${\tau }_{n}$ ($n=1, \cdots , N$) denotes the $n$-th ICI. We conducted the mCSN test to estimate the lower bound ${x}_{{\text{min}}}$ and the power-law exponent $\gamma ,$ while choosing the upper bound as ${x}_{{\text{max}}}=\underset{n}{{\text{max}}}{\tau }_{n}$. We consider the exponential-distribution hypothesis as a competing hypothesis, which is mathematically expressed as follows: $p\left(x\right)={e}^{-\lambda x}/Z\left(\lambda \right)$ $\left({x}_{{\text{min}}}\le x\le {x}_{{\text{max}}}\right)$ with $Z\left(\lambda \right)={\sum }_{x={x}_{{\text{min}}}}^{{x}_{{\text{max}}}}{e}^{-\lambda x}$. Here,${x}_{{\text{min}}}$ and ${x}_{{\text{max}}}$ are the same as those chosen for the power-law fitting. Therefore, let $\widehat{{\varvec{\tau}}}$ be a subset of the ICIs whose lengths are equal to or greater than ${x}_{{\text{min}}}$. Parameter $\lambda$ is estimated by maximizing the log-likelihood:

20a $$\log L^{{({\text{exp}})}} \left( {\widehat{{{\varvec{\uptau}}}};\;\lambda } \right) = \log \prod\limits_{{\tau_{n} \in \widehat{{{\varvec{\uptau}}}}}} {p\left( {\tau_{n} } \right)} = - \sum\limits_{{\tau_{n} \in \widehat{{{\varvec{\uptau}}}}}} {\lambda \tau_{n} } - N\log Z\left( \lambda \right),$$

20b $$\hat{\lambda } = \mathop {\arg \max }\limits_{\lambda } \log L^{{({\text{exp}})}} \left( {\widehat{{{\varvec{\uptau}}}};\;\lambda } \right).$$

We compared the maximum log-likelihoods of the exponential distribution given by Eq. (20) with that for the power-law distribution given by

21a $$\log L^{{({\text{p}}.{\text{l}}.)}} \left( {\widehat{{{\varvec{\uptau}}}};\;\gamma } \right) = - \gamma \sum\limits_{{\tau_{n} \in \widehat{{{\varvec{\uptau}}}}}} {\log \tau_{n} } - N\log Z\left( \gamma \right),$$

21b $$\hat{\gamma } = \mathop {\arg \max }\limits_{\gamma } \log L^{{({\text{p}}{\text{.l}}{.})}} \left( {\widehat{{{\varvec{\uptau}}}};\;\gamma } \right).$$

To show that ${\text{log}}{L}^{({\text{p}}.{\text{l}}.)}\left(\widehat{{\varvec{\uptau}}}; \widehat{\gamma }\right)$ is significantly larger than ${\text{log}}{L}^{({\text{exp}})}\left(\widehat{{\varvec{\uptau}}}; \widehat{\lambda }\right)$, we use the bootstrap method to synthesize $B=100$ pseudo datasets ${\widehat{{\varvec{\tau}}}}_{b}$ $\left(b=1, \cdots , B\right)$ from $\widehat{{\varvec{\tau}}}$. Then, ${\text{log}}{L}^{({\text{p}}.{\text{l}}.)}\left({\widehat{{\varvec{\tau}}}}_{b}; \widehat{\gamma }\right)$ and ${\text{log}}{L}^{({\text{exp}})}\left({\widehat{{\varvec{\tau}}}}_{b}; \widehat{\lambda }\right)$ averaged over the pseudodatasets were compared by calculating their difference ${{\Delta }^{(l.l.)}=\langle {\text{log}}{\widehat{L}}^{\left({\text{p}}.{\text{l}}.\right)}\rangle }_{B}-{\langle {\text{log}}{\widehat{L}}^{\left({\text{exp}}\right)}\rangle }_{B}$, where ${\langle {\text{log}}{\widehat{L}}^{\left({\text{p}}.{\text{l}}.\right)}\rangle }_{B}={\sum }_{b=1}^{B}{\text{log}}{L}^{({\text{p}}.{\text{l}}.)}\left({\widehat{{\varvec{\tau}}}}_{b}; \widehat{\gamma }\right)/B$ and ${\langle {\text{log}}{\widehat{L}}^{\left({\text{exp}}\right)}\rangle }_{B}={\sum }_{b=1}^{B}{\text{log}}{L}^{({\text{exp}})}\left({\widehat{{\varvec{\tau}}}}_{b}; \widehat{\lambda }\right)/B$. The statistical significance of ${\Delta }^{(l.l.)}>0$ was examined using a paired $t$-test.

### Testing independent generation of the ICI series from an identical distribution

Our information-theoretic model also predicts that the ICI series for each dyad is generated independently from an identical power-law distribution. To validate this, we conducted a statistical test to examine whether the autocorrelation was significantly different from zero. The first-order autocorrelation of ${\varvec{\uptau}}$ is given by

22 $$a^{(1)} \left( {{\varvec{\uptau}}} \right) = \frac{{\sum\nolimits_{n = 1}^{N - 1} {\left( {\tau_{n + 1} - \mu } \right)\left( {\tau_{n} - \mu } \right)} }}{{\left( {N - 1} \right)\sigma^{2} }},$$

where $\mu \equiv {\sum }_{n=1}^{N}{\tau }_{n}/N$ and ${\sigma }^{2}\equiv {\sum }_{n=1}^{N}{\left({\tau }_{n}-\mu \right)}^{2}/N$ are the mean and the variance, respectively^53^. If the ICIs are independently and identically distributed, the autocorrelation theoretically vanishes. However, as the number of ICIs in each dyad is limited (from 20 to 39 ICIs) in the dyads examined, ${a}^{(1)}\left({\varvec{\uptau}}\right)$ takes either a positive or negative value. Therefore, we tested the null hypothesis that ${a}^{(1)}\left({\varvec{\uptau}}\right)$ is approximately zero.

To this end, we generated $B=\mathrm{10,000}$ pseudo series ${{\varvec{\uptau}}}_{b} \left(b=1, \cdots , B\right)$ from ${\varvec{\uptau}}$ by bootstrapping. Each pseudoseries satisfied the independent and identically distributed conditions. We then calculated the first-order autocorrelations ${a}^{(1)}\left({{\varvec{\uptau}}}_{b}\right)$ for these pseudoseries and examined their distributions. The 10% left and 10% right areas of this distribution were selected as rejection areas. The null hypothesis is rejected if ${a}^{(1)}\left({\varvec{\uptau}}\right)$ enters either the left or the right rejection area. If ${a}^{(1)}\left({\varvec{\uptau}}\right)$ entered the rightward rejection area, it was considered significantly positive. A positive ${a}^{(1)}\left({\varvec{\uptau}}\right)$ indicates the tendency of ICIs to become progressively longer or shorter. If ${a}^{(1)}\left({\varvec{\uptau}}\right)$ entered the left rejection area, it was considered significantly negative. A negative ${a}^{(1)}\left({\varvec{\uptau}}\right)$ implies oscillating ICI series. If ${a}^{(1)}\left({\varvec{\uptau}}\right)$ enters neither the rightward nor the leftward rejection areas, the null hypothesis cannot be ruled out. It is unlikely that higher-order autocorrelations are significantly positive or negative. whereas first-order autocorrelation vanishes. Therefore, if the above statistical test does not reject the null hypothesis, we conclude that the ICI series is free from autocorrelation; that is, the ICIs are independently generated from an identical distribution.

### Imbalance in the number of ICIs across dyads and a time-opportunity problem

The last part of this section discusses the statistical adequacy of treating the 2,369 ICI samples collected from all dyads. As pointed out earlier, ~ 48% of the 931 dyads appearing in the dataset MID 4.02 experienced MID only once and therefore provided no ICI samples. Additionally, the number of ICI samples provided by each of the remaining dyads is highly variable (see dyad_ mid.csv and dyad_mid_freq.csv stored at https://github.com/HO299792458/PowerLawICI). Such an imbalance in the number of ICIs across dyads may raise concerns that the 2,369 ICI samples are contaminated by some kind of bias, and the observed power-law property is consequently an artefact.

The number of ICIs provided by dyad $d$ depends on the following three factors: the rate of ICI generation ${\gamma }_{d}$, the lower cutoff ${x}_{{\text{min}},d}$ below which ICIs are absent, and the lifetime of this dyad (equivalent to the upper cutoff ${x}_{{\text{max}},d}$ above which ICIs are absent). If ICIs are generable from a power-law distribution, the power-law exponent determines the rate. Higher the power-law exponent, the more frequent the ICI generation. As a power-law distribution $p\left(x\right)\propto 1/{x}^{\gamma }$ $\left(\gamma >0\right)$ becomes infinity as $x\to +\infty$, the lower cutoff ${x}_{{\text{min}},d}$ also substantially influences the frequency of ICI generation. The rate, as well as the lower cutoff, would vary from dyad to dyad as suggested by the results shown in Table 1 and Fig. 7. These explain why the number of ICI samples provided by each dyad is highly variable. As the power law implies likely occurrence of extremely long ICIs, an ICI of the first-round generation may incidentally exceed ${x}_{{\text{max}},d}$ and consequently no ICI samples would be provided from this dyad. With the fact that a mixture of power-law distributions with different exponents can be well approximated by a single power-law distribution, which we have proved earlier in this section, we argue that sampling ICIs from all dyads is tampered with no explicit bias.

The upper cutoff ${x}_{{\text{max}},d}$, the lifetime of dyad $d$, is also highly variable. For instance, the lifetime of GMY-FRN is 198 years (namely, the full recording period of MID 4.02) whereas that of RUS-UKR (Ukraine) is 24 years. We refer to the imbalance in the lifetime across dyads as a ‘time-opportunity problem’. Even if ICIs in each dyad are generable from a power-law distribution with an infinitely extending tail, a power-low distribution fitting ICIs collected from all dyads should have an upper bound ${x}_{{\text{max}}}$ above which power law fails. This upper bound should appear around a medium of upper cutoffs of individual dyads. Indeed, we have confirmed the existence of such an upper bound (Fig. 3). Thus, the time-opportunity problem does not matter in our analysis. Rather, the appearance of an upper bound supports the plausibility of our power-law hypothesis.

The data of MIDs in politically irrelevant dyads may be dubious, as some conflicts in these dyads may have been overlooked in compiling the data. We have verified in the main text that the dilution of a power-law process also gives a power-law process albeit with a slightly reduced exponent (Fig. 6). Thus, inclusion of politically irrelevant dyads does not introduce any sampling bias.

Finally, we chose seven politically relevant dyads and tested the power-law hypothesis for these dyads separately. These tests are free from the imbalance in the number of ICIs and the time-opportunity problem. Nevertheless, we confirmed the plausibility of the power-law hypothesis for these dyads (Tables 1, 2, Fig. 7, and Fig. 8). These clearly show that the observed power-law property fitting the 2369 ICIs is not an artefact due to the imbalance and time-opportunity problems.

### Supplementary Information

Supplementary Information.

## Data Availability

All data used in this study were created from the file in the following repository according to the methods presented in the paper and/or Supplementary Information: https://github.com/HO299792458/PowerLawICI. All methods needed to evaluate the conclusions in the paper are presented in the paper and/or the Supplementary Information. Additional data and methods related to this paper may be requested from the authors.
