# Supplementary material for: Testing the power-law hypothesis of the interconflict interval
Source: Sci Rep. 2023 Dec 19;13:22686. doi: 10.1038/s41598-023-50002-w (PMC10730599; doi:10.1038/s41598-023-50002-w)
Supplement: Supplementary file 1 — Supplementary Information. [file 41598_2023_50002_MOESM1_ESM.pdf]

Supplementary Information for

Testing the power-law hypothesis of the interconflict interval

Hiroshi Okamoto* *et al.*

*Corresponding author. Email: okamoto@coi.t.u-tokyo.ac.jp

Examining the plausibility of the exponential-distribution hypothesis

In the main text, we demonstrated that, for any of the seven dyads we examined, the power-law hypothesis is more plausible than the exponential-distribution hypothesis for ICIs equal to or larger than the estimated lower bound $\hat{x}_{\mathrm{min}}$ (**Tables 1**, **2** and **Fig. 7**). However, these results do not necessarily exclude the possibility that the exponential-distribution hypothesis is more plausible in another domain (i.e., for another value $\hat{x}_{\mathrm{min}}$ of the lower bound). To examine this possibility, we conducted an mCSN test of the exponential-distribution hypothesis. The results are shown in **Tables S1**, **S2** and **Fig. S1**. Comparing these results with those shown in **Tables 1**, **2** and **Fig. 7**, we conclude that fitting with a power-law distribution is more suitable. For details, see **Discussion** in the main text.

Examining the plausibility of the log-normal distribution hypothesis

A log-normal distribution can mimic a power-law distribution for a relatively large interval. To reject the possibility that a log-normal distribution fits the data better than a pure power-law distribution, we compared the power-law hypothesis with the hypothesis that ICIs follow a log-normal distribution. For this, we used Akaike's information criterion (AIC) to consider the difference in the number of parameters characterising these distributions: $\mathrm{AIC} = -2\hat{L} + 2k$, where $\hat{L}$ is the maximum loglikelihood and k is the number of parameters; $k = 1$ for power-law distributions and $k = 2$ for log-normal distributions. The AIC was significantly lower for power-law distributions than for log-normal distributions (**Table S8**).

The trough in the profile of the p-value of the mCSN test is associated with the WWI and WWII

The profile of the p-value as a function of the upper bound x_{max}, given by conducting the mCSN test applied to 2,369 ICIs compiled from all dyads over the entire period (1816~2014), exhibits a conspicuous trough around 9,000 days (~25 years) (**Fig. 3a**). To confirm that this trough is attributed to the WWI and WWII, when the assumption that the process of conflict occurrence in each dyad is independent of those in other dyads is apparently violated, we removed ICIs supposed to be related to either of the world wars and then re-examined the mCSN test applied to the remaining, 2070 ICI samples. Results obtained are shown in **Fig. S2**. The trough disappeared from the profile of the p-value (**Fig. S2a**), demonstrating that the trough shown in **Fig. 3a** is actually related to the WWI and WWII. For detailed interpretations, see **Discussion** in the main text.

Shift of the power-law exponent over the years

We additionally examined whether the power-law exponent is consistent or changing over the years. To this end, we divided the entire period (1816~2014) into the following eras and then conducted the mCSN test applied to each era: (i) the first half of the 19-th century (1816~1858), (ii) the second half of the 19-th century (1859~1858), (iii) the first half of the 20-th century lasting from 1900 to the end of WWII (1946), (iv) the Cold War era (1947~1989), and (v) the post-Cold War era (1990~the present (2014)). The results shown in **Table S3** and **Fig. S3** demonstrate that the estimated power-law exponent has grown gradually over the last ~200 years. For detailed interpretations of these results, see **Discussion** in the main text.

The timing of onset of interstate conflicts obeys a Poisson process

Richardson's earlier works (Richardson 1945, 1946; Clauset 2018) suggested that the timing of onset of full-scale wars (interstate wars, in our terminology), occurring anywhere in the world, conforms to a Poisson process. To examine whether the timing of onset of interstate conflicts, counted without specifying the dyad, also obeys a Poisson process, we defined the dyad-unconditioned inter-conflict intervals (DUC-ICIs, **Fig. S4**). Considering a possible change in the rate of conflict occurrence over the years, we used the above division of the entire period into the following eras under the supposition that the rate of conflict occurrence is constant in each era: (i) 1815–1858, (ii) 1859–1899, (iii) 1900–1946, (iv) 1947–1989, and (v) 1990–2014. DUC-ICI samples compiled for each era underwent the mCSN test of either the power-law hypothesis or the exponential-distribution hypotheses. The results of the CSN test of the power-law hypothesis are summarized in **Table S4**, **Table S5**, and **Fig. S5**. For any of the eras, the p-value of the CSN test is below the criteria of 0.1 (**Table S4**) and the power-law hypothesis is less suitable than the exponential-distribution hypothesis in the estimated domain (**Table S5**). The results of the CSN test of the exponential-distribution hypothesis are summarized in **Table S6**, **Table S7**, and **Fig. S6**. For two eras ((ii) and (v)), the p-value of the CSN test exceeds the criteria of 0.1, indicating that the exponential-distribution hypothesis is plausible (**Table S6**). For any era, the exponential-distribution hypothesis is more suitable than the power-law hypothesis (**Table S7**). Furthermore, the rate of conflict occurrence, estimated by fitting to either the power-law distribution (**Fig. S5**) or the exponential distribution (**Fig. S6**), grows with time. These results suggest that the DUC-ICI follows is more likely to follow an exponential distribution than a power-law distribution, consistent with the Richardson's suggestion.

Fig. S1.

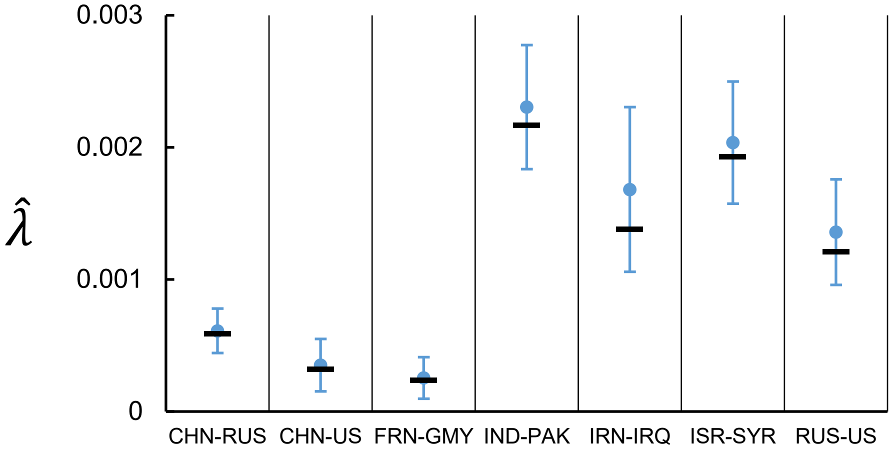

The exponential-distribution parameter λ was estimated for each dyad using the mCSN test. The estimated parameter $\hat{\lambda}$ for each dyad is indicated by the black horizontal bar. To examine the stability of this estimation, 100 pseudo series of ICIs are synthesized by bootstrapping, for each of which the parameter value is re-estimated. The filled blue circle and error bar indicate the mean and standard deviation of these values, respectively.

Fig. S2.

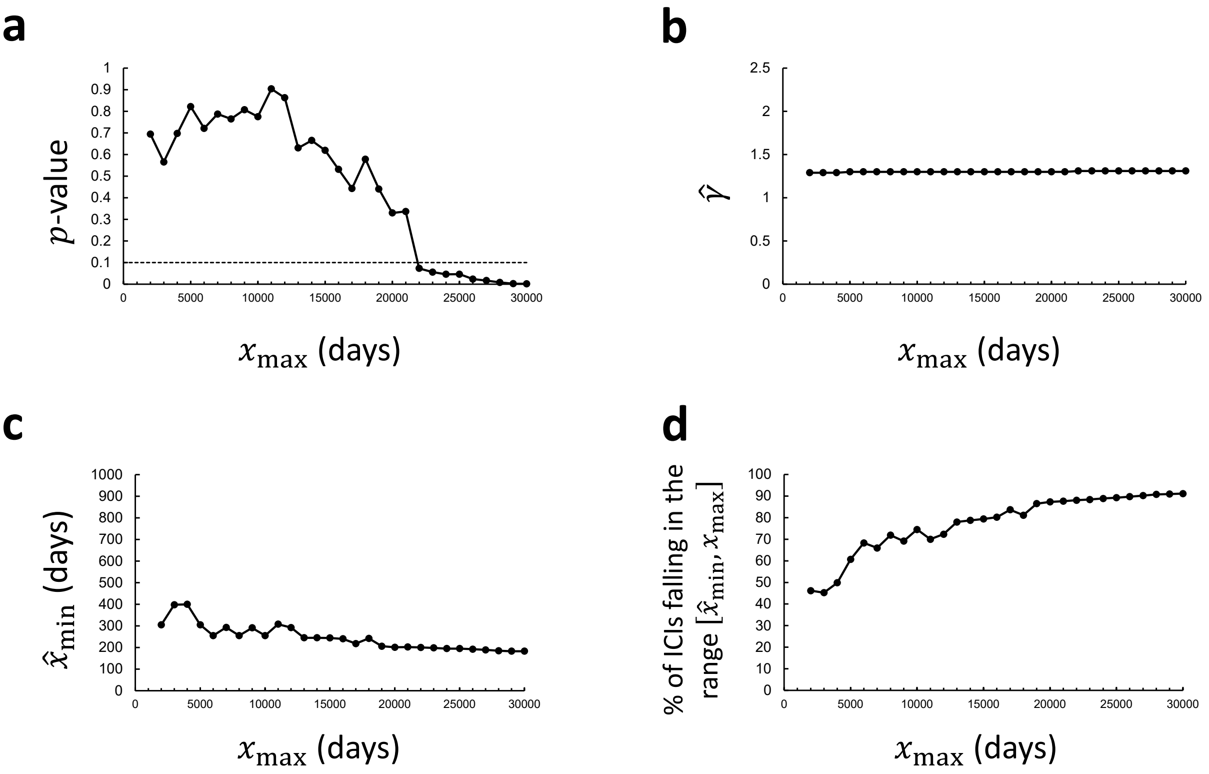

From 2,369 ICI samples collected from all dyads over the entire period (1816~2014), those supposed to be related to the WWI, WWII and interwar period were removed. The same procedures of the CSN test as those for **Fig. 3** were applied to the remaining, 2070 ICI samples. (**a**) The p-value of the mCSN test is plotted as a function of $x_{\max}$. The horizontal dashed line indicates the criterion of 0.1, for the p-value above which the power-law hypothesis is plausible. (**b**) The estimated power-law exponent $\hat{\gamma}$ is plotted as a function of $x_{\max}$. (**c**) The estimated lower-bound $\hat{x}_{\min}$ is plotted as a function of $x_{\max}$. (**d**) The ratio of ICIs (out of the total, 2,369) that fall in the power-law holding domain ($\hat{x}_{\min} \leq x \leq x_{\max}$) is plotted as a function of $x_{\max}$.

Fig. S3.

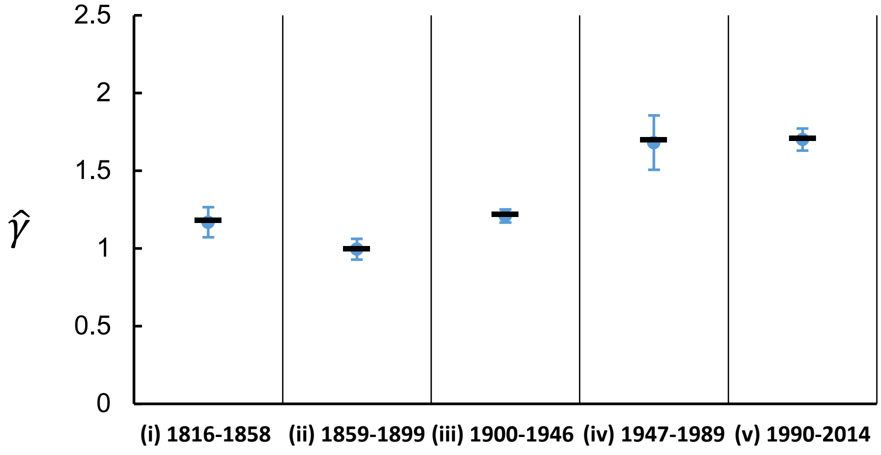

The power-law exponent $\hat{\gamma}$ was estimated for each of the following eras to address whether it is constant or changing over the years: (i) the first half of the 19-th century (1816~1858), (ii) the second half of the 19-th century (1859~1858), (iii) the first half of the 20-th century lasting from 1900 to the end of WWII (1946), (iv) the Cold War era (1947~1989) and (v) the post-Cold War era (1990~the present (2014)). The estimated power-law exponent $\hat{\gamma}$ for each dyad is indicated by the filled black bar. To confirm the stability of this estimation, 100 pseudo-ICI series are synthesized using the bootstrap process, for each of which the power-law exponent is re-estimated. The filled blue circle and error bar indicate the mean and standard deviation of $\hat{\gamma}$.

Fig. S4.

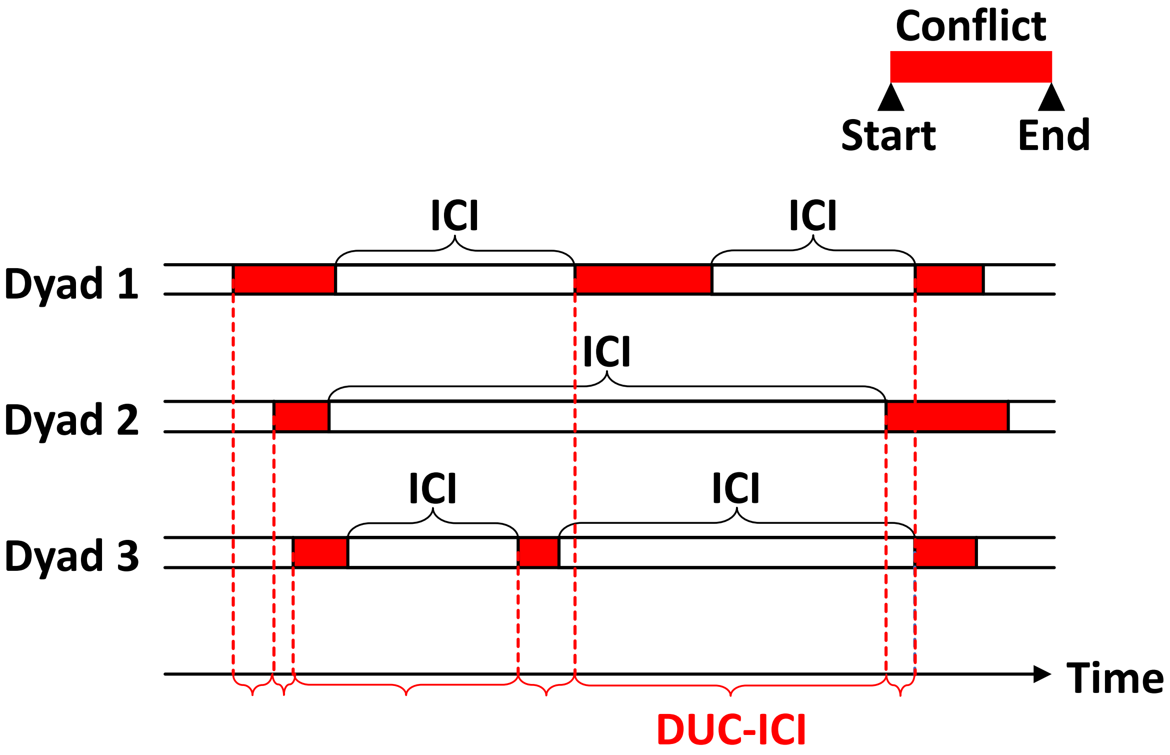

Dyad-unconditioned inter-conflict intervals (DUC-ICIs). The DUC-ICI is the interval between the onset (start) of a conflict and the onset of the next conflict, both counted without specifying the dyad. Each conflict is indicated by the red rectangle.

Fig. S5.

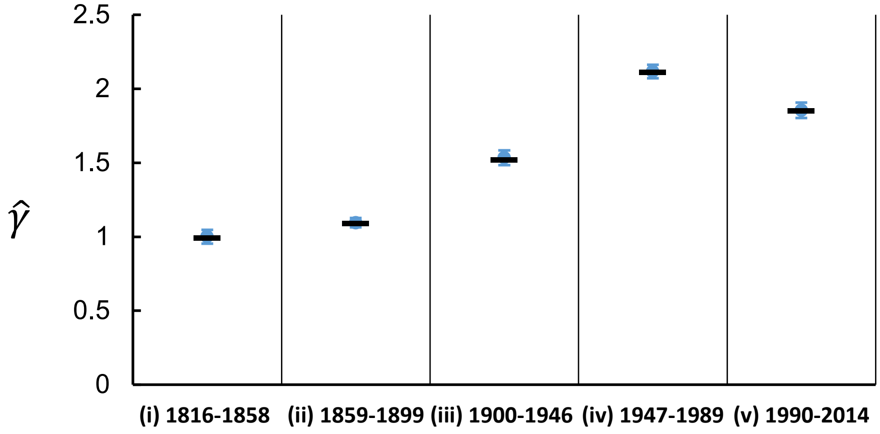

The power-law exponent $\hat{\gamma}$ estimated for the following eras: (i) 1815–1858, (ii) 1859–1899, (iii) 1900–1946, (iv) 1947–1989, and (v) 1990–2014. The power-law hypothesis for the DUC-ICIs was examined for each era using the mCSN test. The estimated power-law exponent $\hat{\gamma}$ for each dyad is indicated by the filled black bar. To confirm the stability of this estimation, 100 pseudo-ICI series are synthesized using the bootstrap process, for each of which the power-law exponent is re-estimated. The filled blue circle and error bar indicate the mean and standard deviation of $\hat{\gamma}$.

Fig. S6.

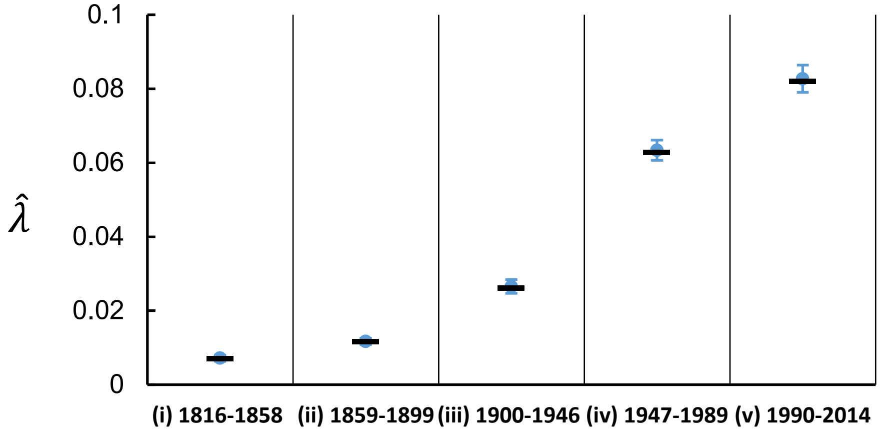

The parameter $\hat{\lambda}$ of the exponential distribution estimated for the following eras: (i) 1815–1858, (ii) 1859–1899, (iii) 1900–1946, (iv) 1947–1989, and (v) 1990–2014. The exponential-distribution hypothesis for the DUC-ICIs was examined for each era using the mCSN test. The estimated parameter $\hat{\lambda}$ for each dyad is indicated by the filled black bar. To confirm the stability of this estimation, 100 pseudo-ICI series are synthesized using the bootstrap process, for each of which the parameter is re-estimated. The filled blue circle and error bar indicate the mean and standard deviation of $\hat{\lambda}$.

Table S1.

	CHN-RUS	CHN-US	FRN-GMY	IND-PAK	IRN-IRQ	ISR-SYR	RUS-US
N	38	29	20	33	29	33	39
$\hat{x}_{\mathrm{min}}$	380	415	379	13	3	3	4
N_{D}	24	14	11	32	29	32	39
N_{D}/N	0.632	0.483	0.550	0.970	1.000	0.970	1.000
$\hat{\lambda}$	0.00059	0.00032	0.00024	0.00217	0.00138	0.00193	0.00121
p-value	0.9172*	0.0012	0.5463*	0.2592*	0.0039	0.176*	0

Results of the mCSN test of the exponential-distribution hypothesis expressed in the form: $p(x) = e^{-\lambda x}/Z(\lambda)$ for $x_{\mathrm{min}} \le x \le x_{\mathrm{max}}$. Here, the value of x_{max} is chosen as the maximum length of ICI samples and the normalization is given by $Z(\lambda) = \sum_{x=x_{\mathrm{min}}}^{x_{\mathrm{max}}} e^{-\lambda x}$. N: the number of ICI samples for each dyad. $\hat{x}_{\mathrm{min}}$: the estimated value of x_{min}. N_{D}: the number of ICI samples within the domain $\hat{x}_{\mathrm{min}} \le x \le x_{\mathrm{max}}$. N_{D}/N: the ratio of ICI samples within the domain. $\hat{\lambda}$: the estimated value of λ. The bottom row lists the p-value of the mCSN test. For the p-value larger than the criteria of 0.1, as indicated by the asterisk (*), the exponential-distribution hypothesis is plausible. The p-value below the criteria is colored red.

Table S2.

	CHN-RUS	CHN-US	FRN-GMY	IND-PAK	IRN-IRQ	ISR-SYR	RUS-US
$\langle \log \hat{L}^{(\text{p.l.})} \rangle_B - \langle \log \hat{L}^{(\text{exp})} \rangle_B$	-0.802	4.391	0.141	-11.371	-10.626	-1.704	-12.962
p-value	1.22E-08	6.38E-39	3.61E-02	1.09E-47	9.49E-23	4.50E-05	1.98E-27

The upper row lists the mean difference $\langle \log \hat{L}^{(\text{p.l.})} \rangle_B - \langle \log \hat{L}^{(\text{exp})} \rangle_B$ for each dyad. The mean $\langle \log \hat{L}^{(\text{p.l.})} \rangle_B$ was calculated by averaging the loglikelihood for the power-law hypothesis over $B = 100$ pseudo series of ICIs generated using the bootstrap process. The mean $\langle \log \hat{L}^{(\text{exp})} \rangle_B$ of the loglikelihood for the exponential-distribution hypothesis was calculated similarly. Positive values of the quantity $\langle \log \hat{L}^{(\text{p.l.})} \rangle_B - \langle \log \hat{L}^{(\text{exp})} \rangle_B$, colored red, indicate that the exponential-distribution hypothesis is less likely than the power-law hypothesis. The bottom row lists the p-value of the paired t-test for each dyad to demonstrate the significance of the negativity or positivity of this quantity.

Table S3.

	(i) 1816-1858	(ii) 1859-1899	(iii) 1900-1946	(iii') 1900-1938	(iv) 1947-1989	(v) 1990-2014
N	64	140	400	252	793	619
$\hat{x}_{\mathrm{min}}$	191	209	205	294	461	540
N_{D}	60	127	355	207	452	310
N_{D}/N	0.938	0.907	0.888	0.821	0.570	0.501
$\hat{\gamma}$	1.18	1	1.22	1.41	1.7	1.71
p-value	0.3383*	0.5603*	0.0286	0.4689*	0.7965*	0.2259*

To address whether the power-law exponent is consistent or changing over the years, we divided the past ~200 years (1816~2014) into the following eras: (i) the first half of the 19-th century (1816~1858), (ii) the second half of the 19-th century (1859~1858), (iii) the first half of the 20-th century lasting from 1900 to the end of WWII (1946), (iv) the Cold War era (1947~1989) and (v) the post-Cold War era (1990~the present (2014)). We then conducted the mCSN test of the power-law hypothesis applied to ICIs collected from each era. We also examined the era, say era (iii'), which is given by trimming the last six years of era (iii) to eliminate the influence of the interwar period. Notations used here are the same as those used for **Table 1**. For the p-value of the mCSN test larger than the criteria of 0.1, as indicated by the asterisk (*), the power-law hypothesis is plausible. The p-value below the criteria is colored red.

Table S4.

	(i) 1816-1858	(ii) 1859-1899	(iii) 1900-1946	(iv) 1947-1989	(v) 1990-2014
N	141	269	787	1300	1052
$\hat{x}_{\mathrm{min}}$	7	6	10	10	7
N_{D}	102	172	338	532	430
N_{D}/N	0.723	0.639	0.429	0.409	0.408
$\hat{\gamma}$	0.99	1.09	1.52	2.11	1.85
p-value	0.0001	0	0.0002	0	0

Results of the mCSN test of the power-law hypothesis for the DUC-ICIs for each of the following eras: (i) 1815–1858, (ii) 1859–1899, (iii) 1900–1946, (iv) 1947–1989, and (v) 1990–2014. Notations used here are the same as those used for **Table 1**. For the p-value of the mCSN test larger than the criteria of 0.1, as indicated by the asterisk (*), the power-law hypothesis is plausible. The p-value below the criteria is colored red.

Table S5.

	(i) 1816-1858	(ii) 1859-1899	(iii) 1900-1946	(iv) 1947-1989	(v) 1990-2014
$\langle \log \hat{L}^{(\mathrm{p.l.})} \rangle_B - \langle \log \hat{L}^{(\exp)} \rangle_B$	-7.558	-25.805	-0.722	-25.784	-29.415
p-value	7.61E-18	9.33E-55	0.226	8.17E-39	8.59E-56

The upper row lists the mean difference $\langle \log \hat{L}^{(\mathrm{p.l.})} \rangle_B - \langle \log \hat{L}^{(\exp)} \rangle_B$ for each era. The bottom row lists the p-value of the paired t-test for each era to demonstrate the significance of the negativity or positivity of this quantity. Notations and calculations used here are the same as those used for **Table 2**.

Table S6.

	(i) 1816-1858	(ii) 1859-1899	(iii) 1900-1946	(iv) 1947-1989	(v) 1990-2014
N	141	269	787	1300	1052
$\hat{x}_{\min}$	6	10	10	9	6
N_{D}	104	157	338	564	468
N_{D}/N	0.738	0.584	0.429	0.434	0.445
$\hat{\lambda}$	0.0071	0.0116	0.0261	0.0628	0.082
p-value	0.0158	0.5546*	0	0.0871	0.7612*

Results of the mCSN test of the exponential-distribution hypothesis for the DUC-ICIs for each of the following eras: (i) 1815–1858, (ii) 1859–1899, (iii) 1900–1946, (iv) 1947–1989, and (v) 1990–2014. Notations used here are the same as those used for **Table S1**. For the p-value of the mCSN test larger than the criteria of 0.1, as indicated by the asterisk (*), the exponential-distribution hypothesis is plausible. The p-value below the criteria is colored red.

Table S7.

	(i) 1816-1858	(ii) 1859-1899	(iii) 1900-1946	(iv) 1947-1989	(v) 1990-2014
$\langle\log\hat{L}^{(\mathrm{p.l.})}\rangle_B - \langle\log\hat{L}^{(\mathrm{exp})}\rangle_B$	-7.076	-25.739	-0.722	-36.635	-36.559
p-value	3.52E-16	8.69E-60	0.226	2.52E-53	3.47E-65

The upper row lists the mean difference $\langle\log\hat{L}^{(\mathrm{p.l.})}\rangle_B - \langle\log\hat{L}^{(\mathrm{exp})}\rangle_B$ for each era. The bottom row lists the p-value of the paired t-test for each era to demonstrate the significance of the negativity or positivity of this quantity. Notations and calculations used here are the same as those used for **Table S2**.

Table S8.

	CHN-RUS	CHN-US	FRN-GMY	IND-PAK	IRN-IRQ	ISR-SYR	RUS-US
$\langle \mathrm{AIC}^{(\mathrm{p.l.})} \rangle_B - \langle \mathrm{AIC}^{(\mathrm{log.n.})} \rangle_B$	-2.5203	-2.368939	-4.222516	-1.618615	-1.155677	-2.119455	-2.129696
p-value	3.65E-29	1.22E-69	1.63E-30	4.28E-41	1.20E-17	1.88E-85	2.28E-40

The upper row lists the mean difference $\langle \mathrm{AIC}^{(\mathrm{p.l.})} \rangle_B - \langle \mathrm{AIC}^{(\mathrm{log.n.})} \rangle_B$ for each dyad. The mean $\langle \mathrm{AIC}^{(\mathrm{p.l.})} \rangle_B$ was calculated by averaging AIC for the power-law hypothesis over $B = 100$ pseudo series of ICIs generated using the bootstrap process. The mean $\langle \mathrm{AIC}^{(\mathrm{log.n.})} \rangle_B$ of the loglikelihood for the log-normal distribution hypothesis was calculated similarly. Negative values of the quantity $\langle \mathrm{AIC}^{(\mathrm{p.l.})} \rangle_B - \langle \mathrm{AIC}^{(\mathrm{log.n.})} \rangle_B$ indicate that the log-normal distribution hypothesis is less likely than the power-law hypothesis. The bottom row lists the p-value of the paired t-test for each dyad to demonstrate the significance of the negativity of this quantity.
